# RING tetramerization is required for nuclear body biogenesis and PML sumoylation

**DOI:** 10.1038/s41467-018-03498-0

**Published:** 2018-03-29

**Authors:** Pengran Wang, Shirine Benhenda, Haiyan Wu, Valérie Lallemand-Breitenbach, Tao Zhen, Florence Jollivet, Laurent Peres, Yuwen Li, Sai-Juan Chen, Zhu Chen, Hugues de Thé, Guoyu Meng

**Affiliations:** 1 State Key Laboratory of Medical Genomics, Shanghai Institute of Hematology, Rui Jin Hospital affiliated to Shanghai Jiao Tong University School of Medicine, 197 Ruijin Er Road, Shanghai, 200025 China; 2 Institute of Health Sciences, Shanghai Institutes for Biological Sciences and Graduate School, Chinese Academy of Sciences, 320 Yueyang Road, Shanghai, 200031 China; 3 University Paris Diderot, Sorbonne Paris Cité, INSERM U944, CNRS UMR7212, Equipe labellisée LNCC, Hôpital St. Louis 1, Paris, 75475 France; 4 Laboratoire International Associé, Hematology and Cancer, RuiJin Hospital, INSERM and CNRS, Shanghai, China; 5 Key Laboratory of Systems Biomedicine, Shanghai Center for Systems Biomedicine, Shanghai Jiao Tong University, 800 Dong Chuan Road, Shanghai, 200240 China; 6 Collège de France, Paris Sciences Lettres research university, 11 place Marcelin Berthelot, 75005 Paris, France; 7 Service de Biochimie, Hôpital St. Louis, Assistance Publique Hôpitaux de Paris, Paris, 75475 France

## Abstract

ProMyelocyticLeukemia nuclear bodies (PML NBs) are stress-regulated domains directly implicated in acute promyelocytic leukemia eradication. Most TRIM family members bind ubiquitin E2s and many acquire ligase activity upon RING dimerization. In contrast, PML binds UBC9, the SUMO E2 enzyme. Here, using X-ray crystallography and SAXS characterization, we demonstrate that PML RING tetramerizes through highly conserved PML-specific sequences, which are required for NB assembly and PML sumoylation. Conserved residues implicated in RING dimerization of other TRIMs also contribute to PML tetramer stability. Wild-type PML rescues the ability of some RING mutants to form NBs as well as their sumoylation. Impaired RING tetramerization abolishes PML/RARA-driven leukemogenesis in vivo and arsenic-induced differentiation ex vivo. Our studies thus identify RING tetramerization as a key step in the NB macro-molecular scaffolding. They suggest that higher order RING interactions allow efficient UBC9 recruitment and thus change the biochemical nature of TRIM-facilitated post-translational modifications.

## Abstract (web summary)

Promyelocytic leukemia protein (PML) is a scaffolding protein that organizes PML nuclear bodies. Here the authors present the tetrameric crystal structure of the PML RING domain and show that RING tetramerization is functionally important for nuclear body formation and PML sumoylation.

## Introduction

PML nuclear bodies (NBs) are membrane-less insoluble structures whose assembly increases upon stress and which recruit a large number of partner proteins^1^. PML NBs also recruit enzymes implicated in several post-translational modifications, primarily UBC9, the key E2 SUMO-conjugating enzyme, possibly facilitating partner sumoylation^2,3^. There is evidence that PML NBs regulate stress responses, in particular senescence induction, at least in part through the control of p53 activation^4–6^. Indeed, NBs are directly implicated in the eradication of acute promyelocytic leukemia (APL) by retinoic acid and arsenic therapy, notably by inducing p53-mediated senescence^7–9^. PML belongs to the TRIM family of proteins, defined by the presence of a RING domain, one or two other zinc fingers (B boxes) and a long coiled coil^10^. Many TRIMs are ubiquitin E3 ligases, through their ability to bridge RING-binding E2 enzymes to specific substrates^11^. Furthermore, TRIM RING dimerization is often required for E2 interaction^12–14^. Interestingly, PML RING interacts with UBC9^15^. Similar to PML, several TRIM family members are interferon-induced, regulate innate immunity and/or assemble into large nuclear or cytoplasmic complexes^12,14^. PML RING, B boxes and coiled coil are all important for NB-biogenesis^16–18^. Although PML sumoylation was proposed to drive PML NB-biogenesis^19,20^, SUMO is required for NB client protein recruitment rather than PML self-assembly into NBs^2,21–23^.

Here we report the crystallographic high-resolution structure of PML RING, and demonstrate that it assembles into a tetrameric torus. Interfaces of these tetramers involve PML-specific sequences that are highly conserved during evolution. Critically, their mutation prevents PML tetramerization in solution and abolishes NB formation and PML sumoylation in cellulo. Our data suggest that this novel macro-molecular RING assembly may control interactions with E2s and hence TRIM functions.

## Results

### RING crystal structure reveals a torus-shaped tetramer

**Fig. 1:**
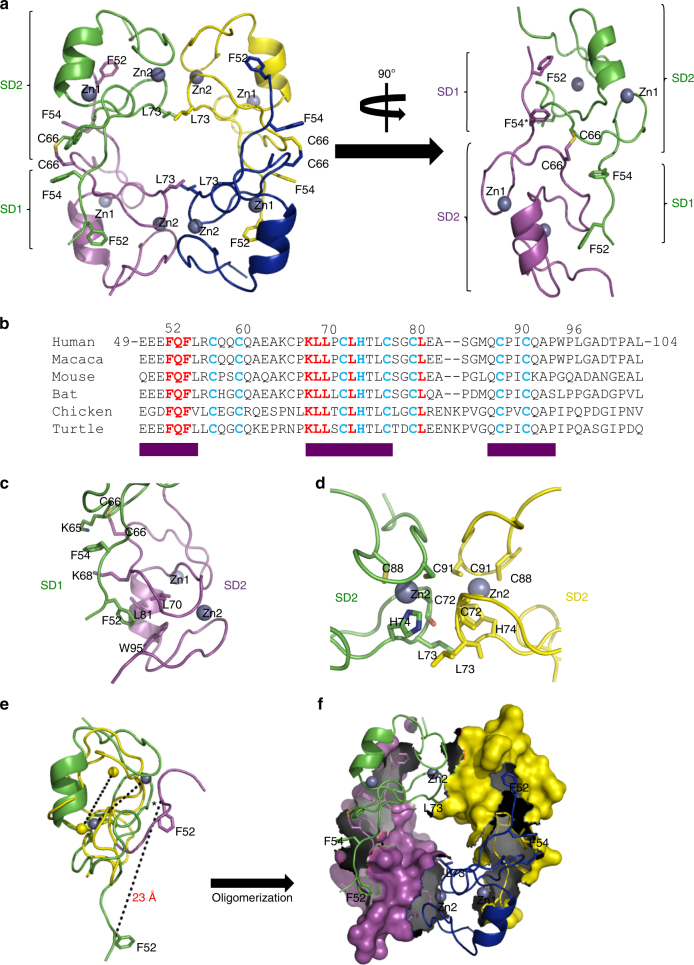
Crystal structure of PML RING tetramer. **a** Crystal structure of PML RING tetramer. The residues 51–97 of crystallized PML RING49–104 are visible in the electron density map. Four PML monomers are colored in green, magenta, blue and yellow, respectively. The contact residues (F52, F54 and L73) are shown in stick representation. Zn ions are shown in sphere representation. Sub-domain 1 (SD1_FQF_) and sub-domain 2 (SD2) are bracketed. **b** Sequence alignment of the PML RING domains from different species. The conserved residues lying in the F52/54-interfaces and L73-interfaces are highlighted in red, while the conserved Zn-binding residues are colored in cyan. The deep purple boxes underneath the sequences are used to highlight the conserved (greater than 5 out of 6) residues among PML RING. **c**, **d** Enlarged views of PML RING dimeric interfaces. The residues involving PML oligomerization are shown in stick representation. **e** Structural superimposition between different PML RINGs. The NMR^16^ and crystallographic PML RINGs are colored in yellow and green, respectively. L73 positions are labeled with “Asterisk”. The internal Zn–Zn distances and the putative F_52_Q_53_F_54_ swing are highlighted with dash lines. **f** The buried areas of the SD1_FQF_/SD2 and SD2/SD2 interfaces are shown in gray

We have determined the crystal structure of PML RING at a 1.6 Å resolution by single wavelength anomalous dispersion (Fig. 1a, Supplementary Figure 1a–e). Similar to previous RING structures^16,24^, the folding of PML RING_49–104_ is coordinated by two Zn ions, but in contrast with previous studies, PML RING leads tetrameric complexes. Within a tetramer, each PML subunit adopts a balloon shape configuration with two distinct sub-domains. Sub-domain 1 (SD1_FQF_) is a loop containing only three residues, F_52_Q_53_F_54_, while sub-domain 2 (SD2), comprising residues 55–99, is a classic C_3_HC_4_ RING finger (Fig. 1a,b). Together with homo-dimerization of SD2, interactions between SD1_FQF_ and SD2 create a four subunits torus-like structure (Fig. 1). Importantly, the residues involved in both interfaces are not only highly conserved among PML orthologs (purple boxes in Fig. 1b), but also highly specific to PML within the TRIM family. The RING tetramer harbors four highly charged patches at each side of the torus as well as a symmetrical deep groove diagonally across each face (Supplementary Figure 1a–e), which could constitute binding sites for partner proteins and/or other PML domains (see below). The interactions between SD1_FQF_/SD2 and SD2/SD2 interfaces of the PML RING tetramer are mediated by: (i) the interaction between F52 from SD1_FQF_ loop of subunit 1, and the hydrophobic pocket delineated by the side chains of L70, L81, W95 from SD2 of subunit 2 (Fig. 1c); (ii) the hydrophobic interactions between F54 from SD1_FQF_, K65 from SD2 of the same subunit 1 and K68 from SD2 of subunit 2, so that the benzyl side chain of F54 is sandwiched by the side chains of K65 and K68 (Fig.1c); (iii) an intermolecular disulfide bridge C66-C66 in the crystal; (iv) two adjacent SD2 subunits pack against each other in a face-to-face configuration mainly mediated by the L73-L73 hand-shake-like hydrophobic interaction, but also by a highly evolutionarily conserved loop around C91 (Fig. 1a,b,d).

When the previous NMR RING structure is superimposed with the crystallographic one, significant differences appear (Fig. 1e). In the previously described monomeric structure, SD1_FQF_ was proposed to interact with the SD2 of the same subunit. Within the current tetramer, the N-terminal F_52_Q_53_F_54_ SD1_FQF_ undergoes a radical 23 Å swing away from the adjacent SD2, allowing the SD1_FQF_ loop to engage with the hydrophobic pocket of the other subunit. This shift also exposes L73 and allows SD2 dimerization (Fig. 1e,f). As estimated by the AREAIMOL^25^ program, the buried surfaces between one set of SD1_FQF_/SD2 and SD2/SD2 dimers are 1333 and 779 Å^2^, respectively (Fig. 1f). As oligomerization proceeds, the buried surface of PML RING tetramer increases significantly to 4216 Å^2^, accounting for ~40% of the overall surface of PML RING tetramer (Fig. 1f).

### Tetramerizationin solution through highly conserved sequences

**Fig. 2:**
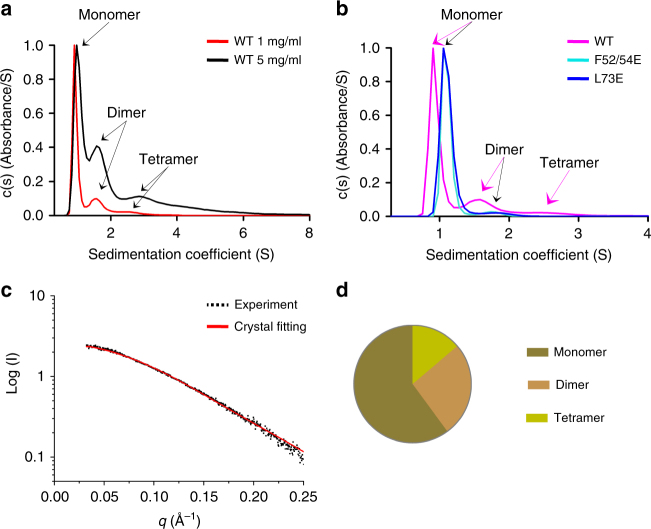
Characterization of PML RING tetramers. **a** Analytical ultracentrifugation analysis of tetramerization of wild type PML RING, at 1 and 5 mg ml^−1^ concentrations, respectively. **b** Analytical ultracentrifugation analysis of 1 mg ml^−1^ PML RING, PML RING_F52/54E_ and PML RING_L73E_. **c** SAXS characterization. Black dots, experimental data. Red line, the theoretical scattering pattern derived from mixs of PML RING multimers. **d** Oligomeric distribution estimated by SAXS analysis. The distribution of PML RING monomer (60.1%), dimer (26.1%) and tetramer (13.8%) was estimated using the OLIGOMER algorithm

Existence of PML RING tetramers is supported by biochemical analyses: analytical ultracentrifuge experiments of PML RING_49–104_ showed three peaks corresponding to molecular masses of 7.5, 15 and 34 kDa, consistent with RING monomer, dimer and tetramer formation (Fig. 2a). Importantly, disruption of either interfaces abolished complex formation (Fig. 2b), while not altering the RING overall fold (Supplementary Figure 2). In order to further document PML RING tetramerization in solution, we used small-angle-X-ray scattering (SAXS) (Fig. 2c). The match in crystal fitting with a *Χ*^2^ value of 1.19 suggested the existence of PML RING monomer (60.1%), dimer (26.1%) and tetramer (13.8%) in solution (Fig. 2d). We then performed gel filtration of PML deleted for B boxes and coiled coil domains (∆BC) to prevent oligomerization, with or without F52/54E or L73E, or with mutations of the SD1_FQF_-interacting amino acid K65, K68 and L81 in SD2 (Supplementary Figure 4a). These PML mutants were consistently found in lower molecular weight fractions compared to PML∆BC, suggesting that they lost their self-interaction properties. Similarly, when the F52/54E mutant was mixed with PML∆BC, the latter was found in lower molecular weight fractions, suggestive for impairment of its tetramerization (Supplementary Figure 4b).

### Tetramers are also stabilized by TRIM-conserved sequence

**Fig. 3:**
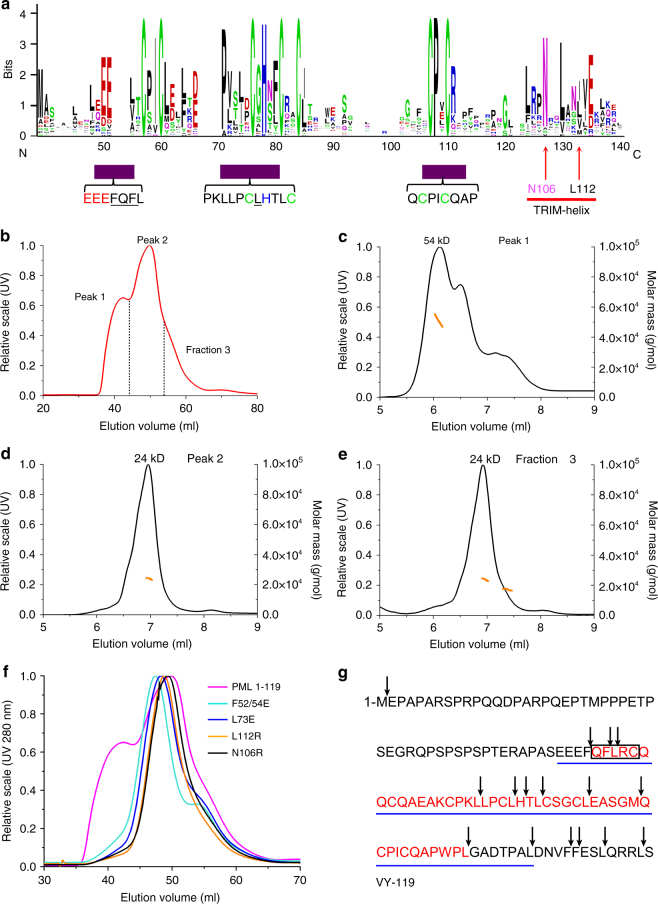
Biochemical evidence for RING tetramer formation. **a** Consensus sequence among TRIM RINGs (Top). PML conserved sequences are highlighted with purple boxes (Bottom). The conserved TRIM dimeric interface is highlighted with a red line. The Asn and Ile/Leu residues (red arrows) mediate dimeric assembly of other TRIMs. **b** Gel filtration analysis of recombinant PML RING_1–119_. Two elution peaks and a last fraction, designated as Peak 1, 2 and fraction 3, respectively. **c**–**e** SEC-MALS reanalysis of Peak 1 (**c**), Peak 2 (**d**) and fraction 3 (**e**) derived from previous gel filtration (**b**). Black curve, the elution profile in UV. Orange curve, the estimated molecular weight. The theoretical molecular weight of PML RING_1–119_ is 13.1 kDa. Peak 1 sample displayed distinct peaks that correspond to the tetramer (54 kDa), dimer and monomer in the solution. Peak 2 and fraction 3 samples primarily exhibit RING dimer (24 kDa). **f** Gel filtration of WT RING_1–119_ and its mutants. **g** Recombinant PML RING_1–119_ was subjected to chymotrypsin digestion (Supplementary Figure 5). Arrows indicate the chymotrypsin sites and amino acids indicated in red correspond to the protected fragment, as determined by mass spectrometry and N-terminal sequencing (Box). RING_49–104_ sequences analyzed by X-ray crystallography are underlined with a blue line

Other TRIM RING domains form dimers rather than tetramers, prompting a detailed analysis of the evolutionary diversity between PML- and TRIM- RING sequences (Fig. 3a and Supplementary Figure 3). Among TRIM RINGs, interactions between N-terminal and C-terminal helix segments were recently implicated in TRIM5, TRIM25 or TRIM32 dimer formation^26–29^. While the N-terminal sequence is not found in PML RING, the highly conserved C-terminal amino acids are present in PML, with N106 and L122 being the two most conserved residues in this TRIM-specific helix (Fig. 3a). An extended PML sequence, RING_1–119_ was thus subjected to gel filtration and SEC-MALS analysis. Critically, we obtained evidence for stable tetrameric and dimeric assembly in solution (Fig. 3b–e). Mutations of either of N106R or L112R inhibited PML RING tetramerization (Fig. 3f). Critically, mutations of residues implicated in the tetramer interfaces (F_52_QF_54_ and L73), which do not alter the RING overall fold (Supplementary Figure 2), also precluded tetramerization of the extended RING (Fig. 3f and Supplementary Figure 4). We finally subjected the long version of PML RING to limited proteolysis and mass sequencing, revealing that residues 53–98 constitute its structured core domain (Fig. 3g and Supplementary Figure 5). Remarkably, this domain is almost identical to the one used for PML RING crystallization, suggesting that the C-terminal helix bundle may somehow be dissociated from the RING core. Collectively, these biochemical studies support the importance of the RING tetramer interfaces for higher order PML RING interactions.

### NB assembly and PML sumoylation requires RING tetramerization

**Fig. 4:**
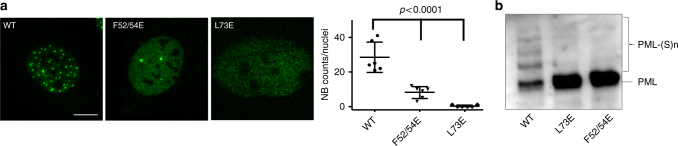
PML tetramerization in NB assembly and PML sumoylation in cellulo. **a** *Pml*^*−/−*^ MEFs stably expressing wild type HA-PML, HA-PML_F52/54E_ and HA-PML_L73E_ were analyzed by immuno-fluorescence using anti-HA antibodies (left, green). Scale bar is 5 μm. Statistical analyses (right). All experiments have been done in five independent replicates, NB count was from 10 to 20 nuclei. Values are means ± S.D. and One-way ANOVA (*p*-values) are indicated. **b** Western blot analysis demonstrating loss of sumoylation of these PML mutants, as detected by anti-HA antibodies. Uncropped images of all Western blots are shown in Supplementary Figures 9–12

To test whether PML RING tetramerization is also important in cellulo, we explored NBs formation and PML sumoylation by stable expression of the mutants in immortalized *Pml*^*−/−*^ fibroblasts (Fig. 4). Critically, these mutants generated significantly fewer NBs (PML_F52/54E_) or even none (PML_L73E_), while the diffuse nuclear staining was dramatically increased. The PML_F52/54A_ mutant behaved as PML_F52/54E_. The effects of single F to E mutations were less drastic, but nevertheless significantly decreased the numbers of NBs (Supplementary Figure 6a). Similarly, combining mutations of the residues facing F52 and F54 on SD2 (K65/68A-L81E and K65/68A-W95E) also reduced NB formation and increased the nuclear diffuse fraction of PML (Supplementary Figure 6d). In contrast, mutations of C66, which is not evolutionarily conserved (Fig.1b), to S or A had modest or no effect on NB biogenesis or basal sumoylation (Supplementary Figure 6d,g). Critically, PML_F52/54E_ or PML_L73E_ failed to undergo efficient sumoylation (Fig. 4b). The more subtle mutants of the SD1_FQF_/SD2 interface showed decreased sumoylation (Supplementary Figure 6b,c,e,f). Mutations in the RING C-terminal conserved TRIM dimeric interface (N106Rand/or L112R, as well as N106A/L112A) also impaired NB biogenesis and sumoylation (Supplementary Figure 6h,i). Collectively, these observations argue that PML RING tetramerization is essential for NB assembly and subsequent PML sumoylation in cellulo.

**Fig. 5:**
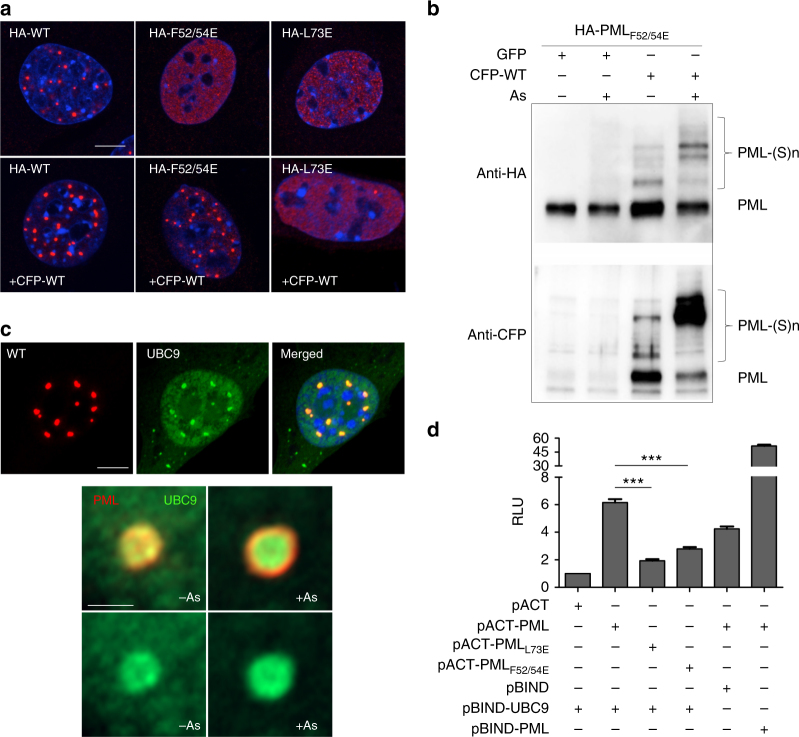
PML tetramerization controls NB formation and PML sumoylation. **a** Immortalized *Pml*^*−/−*^ MEFs were transduced with MSCV virus expressing PML or its mutants and subsequently transduced or not with CFP-PML. PML NBs were monitored by immuno-fluorescence using anti-HA antibody (red). DAPI is in blue. Scale bar is 5 μm. **b** Immortalized *Pml*^*−/−*^ MEFs obtained in **a** were treated with As_2_O_3_ (10^–6^M, 1 h) and extracts were analyzed by Western blot using anti-HA or anti-CFP. PML and its sumoylated forms are indicated. **c** Co-localization of stably expressed HA-PML (red) and UBC9-GFP (green) basal condition (left) or upon 10^−6^M As_2_O_3_ for 1h (right). DAPI is in blue. Middle and bottom: Visualization of PML and UBC9 localization with/without arsenic. Scale bars are 5 μm (top) and 0.5 μm (middle and bottom). **d** Mammalian two-hybrid: relative luciferase activities (RLU) were used to estimate the interaction between UBC9 and PML/mutants. Statistical significance is indicated. All experiments have been done at least with three independent replicates. Values are means ± S.E. ***p < 0.001 are used to show statistically significant between recombinant derivatives. pACT-PML and pBIND-PML interaction is shown as a positive control

*Trans-*complementation assays revealed that co-expression of CFP-WT PML fully restored NBs assembly of HA-PML_F52/54E_, but not that of HA-PML_L73E_ which remained diffusely distributed despite a few aggregates (Fig. 5a and Supplementary Figure 7a). This difference in NB re-assembly suggests that the two L73-L73 interactions are required to stabilize the tetrameric torus, while fewer than four SD1_FQF_/SD2 interfaces may be sufficient. PML RING interacts with UBC9 and PML NBs efficiently recruit UBC9, especially under arsenic-induced oxidative stress^2,15^ (Fig. 5c). Critically, in our *trans-*complementation assays, CFP-WT PML co-expression restores efficient basal or arsenic-enhanced sumoylation of HA-PML_F52/54E_, but never that of PML_L73E_, mirroring NB-reformation (Fig. 5b and Supplementary Figure 7b). We then performed mammalian two-hybrid experiments with either full-length PML or its RING domain. Both demonstrated interaction of PML RING with UBC9, but not RING tetramer mutants (Fig. 5d and Supplementary Figure 7e). The rare NBs formed by PML_F52/54A_ did colocalize with UBC9, but FRAP experiment demonstrated a decreased time of half-recovery, suggesting thatdecreased affinity of this PML mutant with UBC9 may explain its inefficient SUMO-conjugation (Supplementary Figure 7c,d). Collectively, these observations support a role of RING tetramers for NB formation, direct or indirect UBC9 recruitment and PML sumoylation.

### APL development and arsenic response require tetramerization

**Fig. 6:**
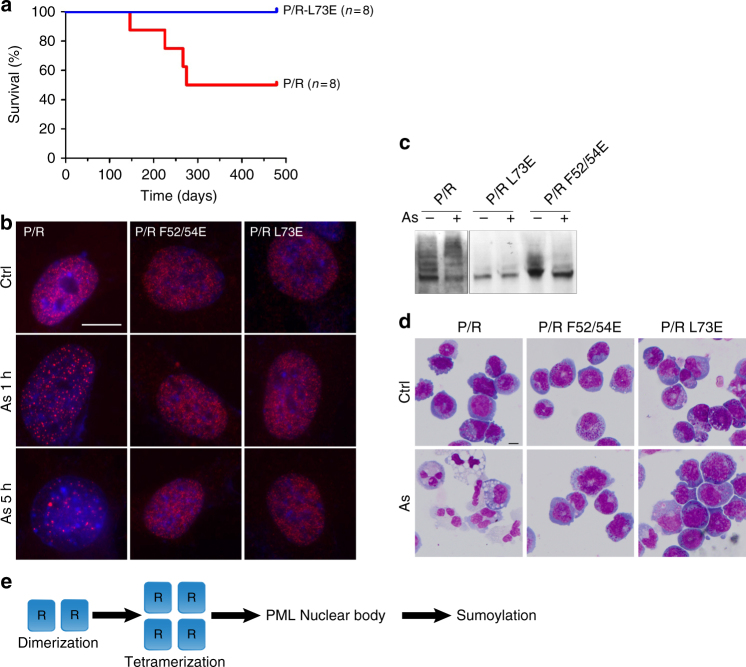
PML RING tetramerization is important for APL development and arsenic targeting of PML/RARA-transformed cells. **a** Survival data of MRP8-PML/RARA or -PML/RARA_L73E_ transgenic mice^42^. **b** *Pml*^*−/−*^ MEFs expressing HA-PML/RARA, HA-PML/RARA_F52/54E_ or HA-PML/RARA_L73E_ were treated with As_2_O_3_ (10^–6^M) prior to immunofluorescence analysis. Scale bar is 5 μm. **c** Sumoylation of HA-PML/RARA after 1 h of As_2_O_3_ exposure was monitored by Western blot using anti-RARA antibody^41^. **d** MGG staining of mouse hematopoietic progenitors transformed by PML/RARA and the indicated mutants after arsenic treatment (10^–7^M, 7 days). **e** Proposed model for NB assembly and PML sumoylation integrating the PML RING tetramer formation

Using PML/RARA transgenic mice, we investigated whether PML RING tetramerization is important in APL development. PML/RARA, but not PML/RARA_L73E_, efficiently developed APL (Fig. 6a), in line with our observation that sumoylation of PML moiety is required for efficient leukemogenesis in vivo^30^. Previous studies have shown that the therapeutic effect of arsenic in APL is based on its ability to trigger PML or PML/RARA re-assembly in NBs, hyper-sumoylation and subsequent PML/RARA degradation^7,21,31–33^. Arsenic was unable to trigger NBs re-assembly and sumoylation of either PML/RARA_F52/54E_ or PML/RARA_L73E_ in *Pml*^*−/−*^ MEFs (Fig. 6b,c). PML/RARA_F52/54E_ or PML/RARA_L73E_ efficiently transformed primary progenitors ex vivo—as assessed by increased clonogenic activity in semi-solid cultures. Yet, the mutants failed to undergo any arsenic-triggered NB re-assembly and terminal differentiation (Fig. 6d and Supplementary Figure 8a,b). Collectively, these data establish the requirement of RING tetramerization in APL development in vivo and arsenic response ex vivo.

## Discussion

PML RING, B boxes and coiled coil domains cooperate to assemble the insoluble NB scaffolds. We discovered that PML RING assembles into tetrameric torus whose formation requires highly evolutionarily conserved PML-specific residues located on the two contact points (Fig. 1b). Their mutations, which do not alter RING folding, abolish PML NB assembly, demonstrating the role of tetramers in higher order of macromolecular assembly (Figs. 2–4). These are likely stabilized by the C-terminal helix bundles, common to the RINGs of other TRIMs. Within the tetramer, L73 is absolutely essential, while the four SD1_FQF_/SD2 contacts quantitatively modulate NB-formation and sumoylation, most likely through tetramer stability (Fig. 5 and Supplementary Figure 7a). Conformation of the extended loop containing SD1_FQF_ could be regulated by phosphorylation of multiple serine residues in the uncommonly long PML N-terminus^34^ (Fig. 1b and Supplementary Figure 3). PML RING tetramers constitute essential building blocks within larger macro-molecular scaffolds, directly or indirectly ensuring efficient UBC9 recruitment. PML sumoylation does not contribute to NB-biogenesis^2^. RING K65 sumoylation is unessential for NB-biogenesis (Supplementary Figure 6d), but could stabilize UBC9 binding^2^ and favor its activity towards other PML sites, notably K160. Arsenic, by targeting the B2 box^35^, accelerates PML NB formation and subsequent sumoylation, a process abolished when the torus interfaces are disrupted (Figs. 5,6 and Supplementary Figure 6,7). The fact that wt PML not only rescues PML_F52/54E_ NB formation but also its sumoylation supports the idea that PML sumoylation occurs in trans within mixed tetramer. Other TRIM proteins are endowed with RING-dependent E3-ubiquitin-ligase activities, dependent on their dimerization^12–14^. We propose that higher order PML RING assembly has endowed PML with the ability to yield NBs, recruit UBC9 and promote PML sumoylation, thus contributing to the functional diversification of the biochemical activity of TRIM family members.

## Methods

### Protein expression and purification

The pET32a vector encoding the PML RING_49–104_ domain (amino acids 49–104)^36^ and a longer RING_1–119_ (amino acids 1–119) were transformed into *Escherichia coli* BL21 (DE3) cells (Sangon) for protein production. The design of primer used in this study is shown in Supplementary Table 1 and 2. The recombinant protein containing a N-terminal cleavable (His)_6_ tag was induced with 200 μM IPTG (Sangon) and 20 μM ZnCl_2_ (Sangon) when the reading of OD_600_ reaches 0.8. The cells were grown at 22 °C for 14 h before harvest by centrifugation (4700*g*, 20 min).

The bacterial cells were resuspended in buffer containing (20 mM Tris, 100 mM NaCl, pH 8.0) and lysed using a cell cracker (JNBIO) applying (20 kg cm^−2^) pressure. Cell debris was removed by centrifugation and clean lysate was loaded onto a pre-equilibrated nickel sepharose column (His Trap HP, GE Healthcare). The column was washed with buffer containing (20 mM Tris, 20 mM Imidazole, 500 mM NaCl, pH 8.0) and PML RING was eluted with buffer containing (20 mM Tris, 150 mM Imidazole, 100 mM NaCl, pH 8.0). The TRX-His tag was removed by digestion with Thrombin enzyme at room temperature overnight after the eluate was dialyzed against Thrombin digestion buffer (Sigma). The cleaved TRX-His tag and uncleaved PML RING were removed by recycle over a pre-equilibrated nickel column. PML RING was purified further with an anion exchange sepharose column (Q HP, GE Healthcare) and a hydrophobic interaction sepharose column (Phenyl HP, GE Healthcare). The correct mass of the protein was confirmed by MS analysis, and the purity was checked by SDS–PAGE.

### Crystallization and data collection

PML RING crystals were grown in 48-well plates using the vapor diffusion technique. Then 0.5-μl PML RING (26 mg ml^−1^) was mixed with 0.5 μl reservoir solution (500 mM Ammonium Sulfate, 1 M Lithium Sulfate and 100 mM Sodium Citrate), and the plates were incubated at 4 °C for ~2 weeks. The crystals were stabilized in a 50:50 mixture of paraffin and paratone-N (Hampton Research), and then were flash-cooled in liquid nitrogen. Diffraction data were collected in Beamline station BL17U at Shanghai Synchrotron Radiation Facility (SSRF, Shanghai, China).

### Phasing and structure refinement

The diffraction data were recorded at the wavelength of Zn anomalous dispersion peak (1.2824 Å) and subsequently processed, integrated and scaled using MOSFLM/SCALA^25^. The statistics of the data collection are shown in Supplementary Table 1. Single wavelength dispersion (SAD) method implemented in CRANK2^25^ was used to phase PML RING. Eight Zn^2+^ positions were determined by CRUNCH2^25^ using data between 20 and 1.6 Å. An interpretable map was obtained by solvent flattening using program SOLOMON^25^. The autotracing program ARP/wARP was then used to produce a σ_A_-weighted 2Fo-Fc map for further manual model building. REFMAC5^37^ was used for structural refinement. Intermittent manual building implemented in COOT^25^ was used to correct and improve the initial models produced by ARP/wARP^38^. The B-factors were refined with TLS corrections (4 TLS group, 84 parameters)^37^. The final model of PML RING tetramer contains 187 residues and 224 water molecules. Ramachandran statistics of PML RING calculated by PROCHECK^39^ indicate that 99.4% of the atoms are in the most favored region, and 0.6% are in the allowed regions. The detailed structure refinement statistics are reported in Table 1.

**Table 1:** Data collection and structure refinement statistics of PML-RING

Data collection	
Space group	P2_1_2_1_2_1_
Unit cell dimension (Å)	
a	38.5
b	84.7
c	86.1
Molecule per ASU	4
Derivative	Native
Source/Station^a^	BL17U
Wavelength (Å)	1.2824, Zn_peak_
Resolution range (Å)	60.4 - 1.60
Observations (*I/*σ(*I*) > 0)	467248
Unique reflections (*I/*σ(*I*) > 0)	37754
High resolution shell (Å)	1.69-1.60
* R*_sym_ (%)^b,c^	14.9 (133.6) < *I/*σ(*I*) > ^c^: 8.0 (1.3)
Completeness^c^ (%)	99.4 (99.9)
Redundancy^c^	2.4 (11.5)
CC_1/2_	0.996 (0.848)
Structure refinement	
Resolution range (Å)	60.4 - 1.60
* R*-factor (%)	20.3
* R*-factor (high resolution shell)^d^	35.0
* R*_free_ (%)^e^	21.7
* R*_free_ (high resolution shell)	36.6
Total number of non-hydrogen atoms	
Protein atoms	1403
Water molecules	224
Zn ions	8
R.m.s. deviations^f^	
Bond length (Å)	0.005
Bond angle (°)	0.954
Main chain *B*-factors (Å^2^)	1.799
Side chain *B*-factors (Å^2^)	5.871
Wilson *B*-factor (Å^2^)	22.1
Average *B*-factor (Å^2^)	
Protein atoms	43.8
Solvent atoms	49.5
Zn ions	38.3
Ramachandran statistics (%)	
Most favored region	99.4
Allowed regions	0.6

^a^Beamline designations refer to the Shanghai Synchrotron Radiation Facility, Shanghai, P. R. of China

^b^*R*_sym_ = Σ(*I *− <*I*>)^2^/Σ*I*^2^

^c^overall, high resolution shell in parentheses

^d^high resolution shell: 1.640–1.600 Å

^e^*R*_free_ calculated using 5% of total reflections omitted from refinement

^f^R.m.s. deviations report root mean square deviations from ideal bond lengths/angles and of *B*-factors between bonded atoms^44^

### Analytical ultracentrifugation and gel filtration analysis

Sedimentation experiments were conducted using a Beckman XL-I Optima analytical ultracentrifuge equipped with absorbance optics. Sedimentation studies were carried out at 200,000*g*, at 25 °C overnight. Three-channel with quartz windows were filled with 400 μl of sample (20 mM Tris, 100 mM NaCl, pH 8.0, with/without 1 mM DTT) at the concentration of 1 mg ml^−1^. To investigate how protein concentration might influence PML RING tetramerization, the wild type protein at the higher concentration (5 mg ml^−1^) was also tested. Absorbance profiles were acquired at a wavelength of 335 nm, chosen according to the protein concentration. Data analysis was carried out using SEDFIT^40^, which employs the continuous c(s) conformational change model based on the Lamm equation, to determine the sedimentation coefficient distribution.

In order to check RING tetramerization in the context of full-length PML protein, gel filtration analysis was used. Recombinant proteins including PML∆BC (i.e., the deletion of residues, 120–360) and mutants were obtained by in vitro translation (Promega) using pcDNA3.1(−) vector with T7 promoter according to manufacturer’s standard protocol. The sample was then subjected to gel filtration analysis using Superose 6 and Superose 12 columns (GE) at a flow rate of 0.4 ml min^−1^. Each fraction was monitored by Western blot using antibody against PML (Abcam).

### Cell culture and treatments

For expression in MEFs or progenitors, MSCV retroviral constructs were used, HA or CFP tags were in frame with 5′ PML coding sequence. Mutations were generated with the QuikChange II site-directed mutagenesis kit (QIAGEN) on MSCV-HA-His_6_-PML or pSG5-HA-His_6_-PML and subcloned in MSCV-HA-His_6_-PML (named HA-PML) or in MSCV-HA-His_6_-PML/RARA (HA-PML/RARA). Oligonucleotides used in all the constructs arelisted in Supplementary Table 2.

Immortalized *Pml*^*−/−*^ MEF cells, obtained previously by large T expression in primary MEFs^35^, were transduced with MSCV virus expressing HA-His_6_-tagged WT or mutants. For rescue experiments, MEFs stably expressing HA-PMLs were transduced with MSCV-CFP-WT PML or MSCV-GFP. Immortalized *Pml*^*−/−*^ MEF stably expressing HA-WT PML (or indicated mutants) were transduced with MSCV virus expressing UBC9-GFP as described in the figures. MEFs were treated with 10^–6^ M As_2_O_3_ (Fluka) for 1 h before protein extraction and Western blot analysis using anti-HA, anti-CFP, anti-GFP, and anti-Lamin antibodies.

Mouse haematopoietic progenitors (i.e., lineage-depleted bone marrow from 5-fluorouracil-treated C57BL/6 mice) were transduced with MSCV-HA-His_6_-PML/RARA or its mutants, cultured in RPMI medium supplemented with IL-3, IL-6 and stem cell factor and treated with 10^–6^ M As_2_O_3_ for 1 h. For FACS and MGG experiments, transduced cells were re-plated in methylcellulose at a density of 10,000 cells/dish in the presence or absence of 10^–7^ M As_2_O_3_. Cells were analyzed after 7 days using FACS according to manufacturer’s guidelines and MGG staining.

### Immunoflorescence image acquision and Western blot

Cells were fixed with 4% paraformaldehyde. Immunofluorescence assays were performed and analysed by confocal microscopy using the antibodies described below. The slides were examined with a Leica TCS SP8 or Zeiss LSM870 confocal fluorescent microscope. Protein extracts were prepared by lysing cells directly in Laemmli buffer. SUMO conjugates and PML proteins were separated on 4–12% gradient SDS–PAGE (Biorad). Homemade chicken polyclonal anti-human PML was previously described^41^. Mouse monoclonal anti-HA was from Covance, anti-GFP from Roche, rabbit polyclonal anti-CFP and goat polyclonal anti-lamin B antibodies from Sigma-Aldrich. Anti-mouse cKit (CD117) and anti-mouse Mac1 antibodies were from BD pharmingen. Alexa 488- or 594-labeled secondary antibodies and HRP-conjugated secondary antibodies from Jackson Laboratories.

For statistics, PML NBs were counted from at least 50 randomly chosen cells. The ratio between sumoylated and unmodified PML was calculated from Vilber Lourmat Fusion camera software. All experiments have been done at least with three independent replicates.

### FRAP analysis

Fluorescence recovery after photobleaching (FRAP) was performed on Zeiss LSM510 confocal microscope equipped with a heated chamber. MEF cells expressing HA-PMLwt or HA-PML_F52/54A_ together with UBC9-GFP were seeded in glass-bottomed dish in HEPES-buffered DMEM media with 10% FBS and the dish was mounted on the stage in the confocal chamber pre-heated to 37 °C. The cells were observed under a 63× oil lens. The“region of interest (ROI)”containing only one PML NB was selected, and UBC9-GFP was bleached with a 488 nm argon laser at 100% intensity for 6 times. The means of relative intensity of UBC9-GFP fluorescence during fluorescent recovery were quantified from three independent experiments.

### Transgenic mice

PML/RARA and PML/RARA_L73E_ were expressed using human MRP8 promoter^42^. Mice were housed in specific pathogen-free conditions and all animal experiments were approved by the Animal Care and Use Committee at Experimental Animal Center in Shanghai Jiao Tong University School of Medicine. To define whether the mice have developed APL, hematological disorders such as splenomegaly, MGG staining and flow cytometry analysis of c-Kit, GR1 and Mac-1 in spleen and bone marrow cells were performed.

### Circular dichroism

Samples for CD were prepared by desalting the bacterial expressed recombinant proteins and diluting them to 0.2 mg ml^−1^ using 10 mM CHES buffer (pH 9.0). Far UV CD spectra was recorded from 185 nm to 260 nm at 20 °C with a time constant of 1 s using a Chirascan spectrometer (Applied Photophysics Ltd). The spectra were the average of not less than three scans and presented as mean residue molar ellipticity [θ] (deg.cm^2^ dmol^−1^). Secondary structure content reported in Supplementary Figure 2b was estimated using Circular dichroism (CD) deconvolution program CDNN.

### SEC-MALS analysis

The purified PML RING_1–119_ and mutants were subjected to gel filtration analysis (S100 column, GE Healthcare). The elution peaks, as monitored by UV absorption at 280 nm, were pooled separately and chosen for size exclusion chromatography-multi-angle light scattering **(**SEC-MALS) characterization, respectively. In brief, the purified protein samples were concentrated and analyzed using a WTC-015S5 sized exclusion column (Wyatt Technology) which was connected to a 1260 infinity liquid chromatography system (Agilent Technology) equipped with inline DAWN HELEOS-II MALS and Optilab rEX differential refractive index detectors (Wyatt Technology). For each sample, a 40 μl injection volume and 0.5 ml min^−1^ flow rate were applied. Data were recorded and processed using ASTRA VI software (Wyatt Technology).

### Small angle X-ray scattering

The recombinant PML RING_49–104_ was purified and concentrated to 1, 3 and 5 mg ml^−1^ in 20 mM Tris, pH 8.0, 100 mM NaCl, with/without 1 mM DTT, respectively. The X-ray scattering experiment was carried out at Beamline station BL19U2 (National Facility for Protein Science Shanghai, NCPSS, China) and scattered X-ray intensities were collected by using a Pilatus 1 M detector (DECTRIS Ltd). The measurements were carried out with 1 s exposure time and repeat for 20 times to avoid possible sample radiation damage. The collected data were processed with ATSAS software package^43^. The details of SAXS data collection and processing are shown in Supplementary Table 3. The guinier region of experimental group are linear, indicating the measured sample is homogeneous in solution. Crystal data fitting was done using the OLIGOMER algorism implemented in CRYSOL. In the analysis of crystal fitting (0.01 ≤ *q* ≤ 0.25 Å^−1^), the crystallographic tetramer, crystallographic dimers and crystallographic and NMR monomers were used to fit the merged experimental SAXS data derived from three protein concentrations, leading to the determination of each fraction of monomer/dimer/tetramer in the solutions.

### Mammalian two-hybrid assay

Mammalian two-hybrid assay was performed using the CheckMate^TM^ Mammalian Two-Hybrid System (Promega) in 293 T or CHO cells. The cDNA of UBC9 and full length WT PML or mutants were inserted into pBIND and pACT vectors, respectively. 293T cells were then transfected with plasmids pG5/luc, pBIND-UBC9 and pACT-PML/mutant (mixed at a molar ratio of 1:1:1) using liposome Lipo2000 transfection method (Life Technology). In the complementary set of experiments, UBC9 was cloned into pACT and the extended RING domain into pBIND. The resulting plasmids were transfected in CHO cells using Effecten (Quiagen). Twenty-four hours after transfection, luciferase activity was determined using the Dual-Luciferase Reporter Assay System (Promega).

### Limited proteolysis

10 μg purified PML RING_1–119_ was subjected to chymotrypsin (Sigma) digestion at 25 °C for 20 min. The reaction mixture contained 10 µg RING_1–119_, a series dilutions from 10 μg chymotrypsin, 100 mM Tris (pH 8.0), 10 mM CaCl_2_ and 1 mM DTT. The reaction was terminated by boiling with SDS loading buffer. The sample was analyzed by SDS–PAGE followed by silver staining.

Mass spectrometry analysis was conducted with 5800 MALDI-TOF/TOF (AB Sciex). The limited digestion product was subjected to the PVDF (GE Healthcare) membrane transferring followed by Ponceau S staining to indicate the target protein band. Cropped PVDF membrane containing the target protein was placed into the reactor of the PPSQ-33A (SHIMADZU) automatic protein sequencer, followed by a standard analysis procedure.

### Data availability

Coordinates and structure factors have been deposited in the Protein Data Bank under accession code 5YUF. Other data are available from the corresponding authors upon request.

## Electronic supplementary material

Supplementary Information
