# Publisher Correction: RING tetramerization is required for nuclear body biogenesis and PML sumoylation

**DOI:** 10.1038/s41467-018-04347-w

**Published:** 2018-05-04

**Authors:** Pengran Wang, Shirine Benhenda, Haiyan Wu, Valérie Lallemand-Breitenbach, Tao Zhen, Florence Jollivet, Laurent Peres, Yuwen Li, Sai-Juan Chen, Zhu Chen, Hugues de Thé, Guoyu Meng

**Affiliations:** 1 State Key Laboratory of Medical Genomics, Shanghai Institute of Hematology, Rui Jin Hospital affiliated to Shanghai Jiao Tong University School of Medicine, 197 Ruijin Er Road, Shanghai, 200025 China; 2 Institute of Health Sciences, Shanghai Institutes for Biological Sciences and Graduate School, Chinese Academy of Sciences, 320 Yueyang Road, Shanghai, 200031 China; 3 University Paris Diderot, Sorbonne Paris Cité, INSERM U944, CNRS UMR7212, Equipe labellisée LNCC, Hôpital St. Louis 1, Paris, 75475 France; 4 Laboratoire International Associé, Hematology and Cancer, RuiJin Hospital, INSERM and CNRS, Shanghai, China; 5 Key Laboratory of Systems Biomedicine, Shanghai Center for Systems Biomedicine, Shanghai Jiao Tong University, 800 Dong Chuan Road, Shanghai, 200240 China; 6 Collège de France, Paris Sciences Lettres Research University, 11 place Marcelin Berthelot, Paris, 75005 France; 7 Service de Biochimie, Hôpital St. Louis, Assistance Publique Hôpitaux de Paris, Paris, 75475 France

Correction to: *Nature Communications* 10.1038/s41467-018-03498-0, published online: 29 Mar 2018.

In the originally published version of this Article, the authors Sai-Juan Chen and Zhu Chen were incorrectly listed as being affiliated with ‘University Paris Diderot, Sorbonne Paris Cité, INSERM U944, CNRS UMR7212, Equipe labellisée LNCC, Hôpital St. Louis 1, Paris 75475, France’, and the affiliation ‘Institute of Health Sciences, Shanghai Institutes for Biological Sciences and Graduate School, Chinese Academy of Sciences, 320 Yueyang Road, Shanghai 200031, China’ was inadvertently omitted. These errors have now been corrected in both the PDF and HTML versions of the Article.

