# Supplementary material for: RING tetramerization is required for nuclear body biogenesis and PML sumoylation
Source: Nat Commun. 2018 Mar 29;9:1277. doi: 10.1038/s41467-018-03498-0 (PMC5876331; doi:10.1038/s41467-018-03498-0)
Supplement: Supplementary file 1 — Supplementary Information [file 41467_2018_3498_MOESM1_ESM.pdf]

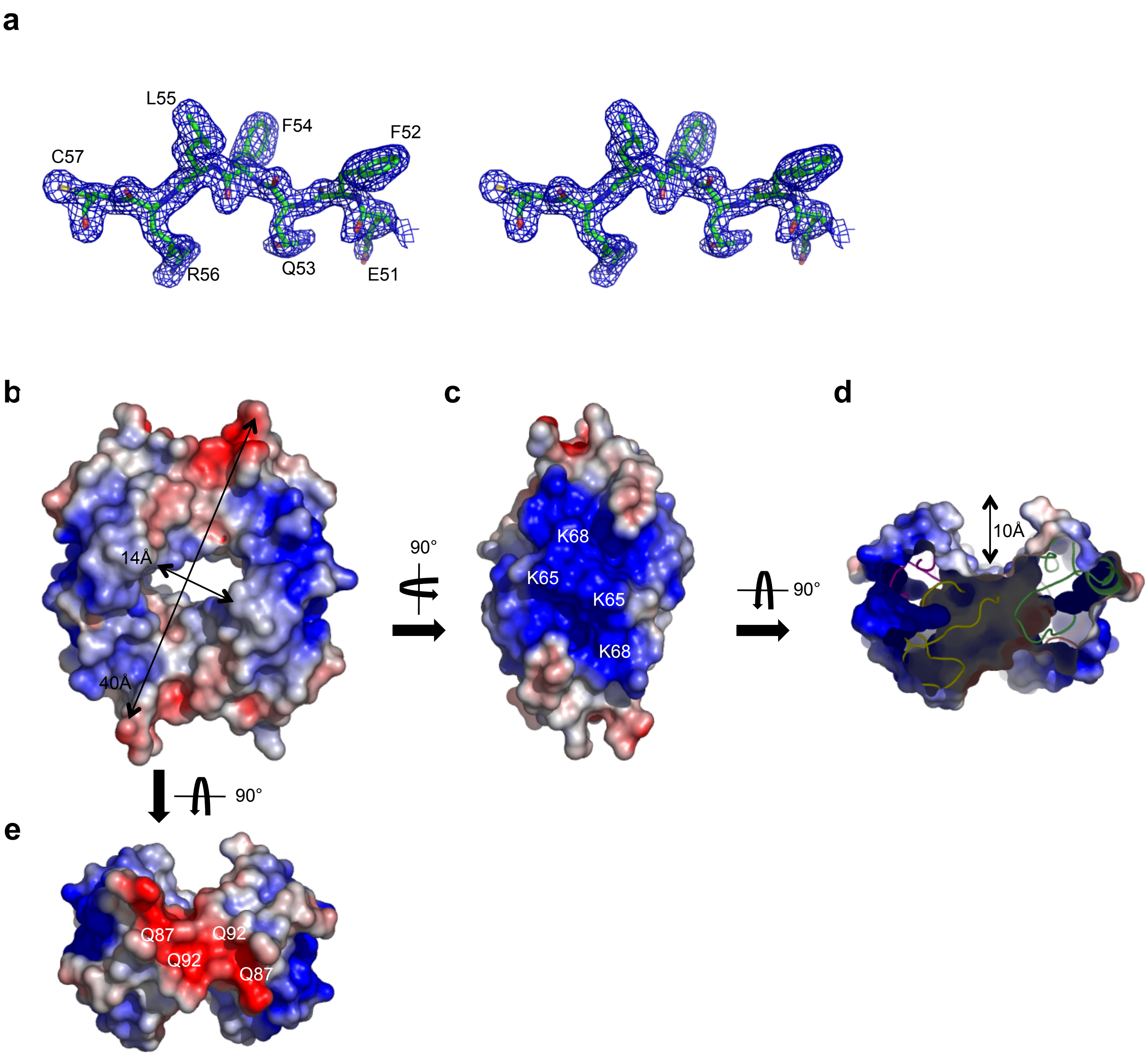

Supplementary Figure 1. The electrostatic surface of PML RING tetramer. a) The stereo image of The σ_A-weighted $2F_O$-F_C density map contoured at 1σ map (blue). b-e) The PML RING tetramerization yields 2 diagonal central grooves (b), 2 highly positively charged K65-K68 pockets (c), and 2 highly negatively charged Q87-Q92 pockets (e). d) Slabbed view of the diagonal groove.

a

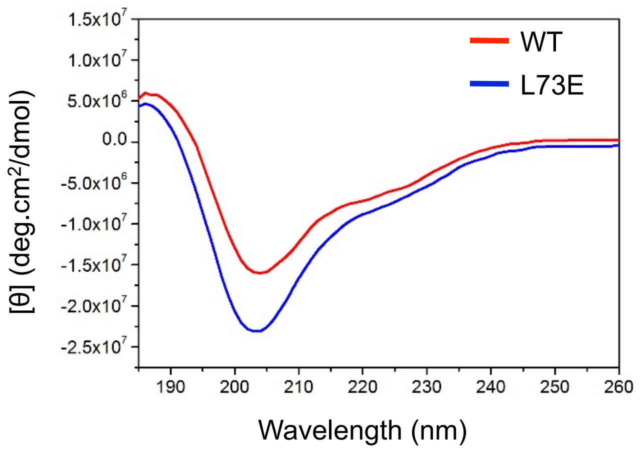

b

Estimate of protein secondary structure

	RING	L73E
wavelength	185-260 nm	185-260 nm
Helix	11.90%	9.20%
Antiparallel	33.10%	36.10%
Parallel	4.10%	3.40%
Beta-Turn	22.10%	23.90%
Rndm. Coil	28.50%	25.50%
Total Sum	99.60%	98.00%

Supplementary Figure 2. Circular Dichroism (CD) characterization of PML RING and mutant. a) CD characterization of PML RING and L73E mutant. b) The statistics of protein secondary structures in PML RING and L73E mutant.

```
PML        1-MEPAPARSPRPQQDPARPQEPTMPPPETPSEGRQPSPSPSPTERAPASEEEFQFLRCQQCQAEAK----CPKLLPCLHTLCSGCLEA---------------SGMQCPICQAPWPLGAD--TPALDNVFFESLQRRLSVY-119
TRIM5        --------------------------------------MASG-ILVNVKEE---VTCPICLELLTQ----PLSLDCGHSFCQACLTANHKKSML-D-----KGESSCPVCRISYQPE-N----IRPNRHVANIVEKLREV
TRIM25       -----------------------------------------MAELCPLAEE---LSCSICLEPFKE----PVTTPCGHNFCGSCLNETWAVQ---------GSPYLCPQCRAVYQARPQ----LHKNTVLCNVVEQFLQA
TRIM32       ----------------------------------MAAAAASHLNLDALREV---LECPICMESFTEEQLRPKLLHCGHTICRQCLEKLLASS---------INGVRCPFCSKITRIT--SLTQLTDNLTVLKIIDTAGLS
TRIM37       ---------------------------------------MDEQSVESIAEV---FRCFICMEKLRDARLC---PHCSKLCCFSCIRRWLTEQ-----------RAQCPHCRAPLQLREL--VNCRWAEEVTQQLDTLQLC
TRIM39       ---------------------MAET-SL---LEAGASAASTAAALENLQVE---ASCSVCLEYLKE----PVIIECGHNFCKACITRWWED----L-----ERDFPCPVCRKTSRYR-S----LRPNRQLGSMVEIAKQL
TRIM56       ---------------------------M---VSHGSSPSL---LEALSSDF---LACKICLEQLRA----PKTLPCLHTYCQDCLAQLAD-----------GGRVRCPECRETVPVPPEGVASFKTNFFVNGLLDLVKAR
TRIM8        --------------------------------------MAE-NWKNCFEEE---LICPICLHVFVE----PVQLPCKHNFCRGCIGEAWAKD---------SGLVRCPECNQAYNQKPG----LEKNLKLTNIVEKFNAL
TRIM13       --------------------------------------------MELLEED---LTCPICCSLFDD----PRVLPCSHNFCKKCLEGILEGSVRNSLWRPA--PFKCPTCRKE--TSATGINSLQVNYSLKGIVEKYNKI
TRIM26       --------------------------------------MATSAPLRSLEEE---VTCSICLDYLRD----PVTIDCGHVFCRSCTTDVRPISG---------SRPVCPLCKKPFKKE-N----IRPVWQLASLVENIERL
TRIM59       --------------------------------------------MHNFEEE---LTCPICYSIFED----PRVLPCSHTFCRNCLENILQASGNFYIWRPLRIPLKCPNCRSITEIAPTGIESLPVNFALRAIIEKYQQE
TRIM1        ---------------------MGESPAS---VVLNASGGLFSLKMETLESE---LTCPICLELFED----PLLLPCAHSLCFSCAHRILVSSCSSGESIEPITAFQCPTCRYVISLNHRGLDGLKRNVTLQNIIDRFQKA
TRIM4        ------------------------------------------MEAEDIQEE---LTCPICLDYFQD----PVSIECGHNFCRGCLHRNWAP----G-----GGPFPCPECRHPSAPA-A----LRPNWALARLTEKTQRR
TRIM6        --------------------------------------MTSP-VLVDIREE---VTCPICLELLTE----PLSIDCGHSFCQACITPNGRESVIGQ-----EGERSCPVCQTSYQPG-N----LRPNRHLANIVRRLREV
TRIM10       --------------------------------------MASAASVTSLADE---VNCPICQGTLRE----PVTIDCGHNFCRACLTRYCEIPGPDL-----EESPTCPLCKEPFRPG-S----FRPNWQLANVVENIERL
TRIM11       --------------------------------------MAAPDLSTNLQEE---ATCAICLDYFTD----PVMTDCGHNFCRECIRRCW----GQP-----EGPYACPECRELSPQR-N----LRPNRPLAKMAEMARR-
TRIM17       --------------------------------------MEAVELARKLQEE---ATCSICLDYFTD----PVMTTCGHNFCRACIQLSWEKARGKKGRRKRKGSFPCPECREMSPQR-N----LLPNRLLTKVAEMAQQ-
TRIM18       --------------------------------------------METLESE---LTCPICLELFED----PLLLPCAHSLCFNCAHRILVSHCATNESVESITAFQCPTCRHVITLSQRGLDGLKRNVTLQNIIDRFQKA
TRIM21       --------------------------------------MASAARLTMMWEE---VTCPICLDPFVE----PVSIECGHSFCQECISQVG------K-----GGGSVCPVCRQRFLLK-N----LRPNRQLANMVNNLKEI
TRIM22       --------------------------------------MDFS-VKVDIEKE---VTCPICLELLTE----PLSLDCGHSFCQACITAKIKESVIIS-----RGESSCPVCQTRFQPG-N----LRPNRHLANIVERVKEV
TRIM31       --------------------------------------MASGQFVNKLQEE---VICPICLDILQK----PVTIDCGHNFCLKCITQIGETS---------CGFFKCPLCKTSVRKN-A----IRFNSLLRNLVEKIQAL
TRIM34       --------------------------------------MASK-ILLNVQEE---VTCPICLELLTE----PLSLDCGHSLCRACITVSNKEAVTSM-----GGKSSCPVCGISYSFE-H----LQANQHLANIVERLKEV
TRIM35       ---------------------------------MERSPDVSPGPSRSFKEE---LLCAVCYDPFRD----AVTLRCGHNFCRGCVSRCWEV----------QVSPTCPVCKDRASPA-D----LRTNHTLNNLVEKLLRE
TRIM38       --------------------------------------MASTTSTKKMMEE---ATCSICLSLMTN----PVSINCGHSYCHLCITDFFKNPSQKQ---LRQETFCCPQCRAPFHMD-S----LRPNKQLGSLIEALKET
TRIM40       ----------------------------------------MIPLQKDNQEE---GVCPICQESLKE----AVSTNCGHLFCRVCLTQHVEKASA-------SGVFCCPLCRKPCSEE-V----L----------------
TRIM50       --------------------------------------MAWQVSLLELEDW---LQCPICLEVFKE----PLMLQCGHSYCKGCLVSLSCH----L-----DAELRCPVCRQAVDGS-S----SLPNVSLARVIEALRLP
TRIM58       --------------------------------------MAWAPPGERLRED---ARCPVCLDFLQE----PVSVDCGHSFCLRCISEFCEKSDGAQ-----GGVYACPQCRGPFRPS-G----FRPNRQLAGLVESVRR-
TRIM60       --------------------------------------MEFVTALVNLQEE---SSCPICLEYLKD----PVTINCGHNFCRSCLSVSWKD----L-----DDTFPCPVCRFCFPYK-S----FRRNPQLRNLTEIAKQL
TRIM62       -------------------------------------------MACSLKDE---LLCSICLSIYQD----PVSLGCEHYFCRRCITEHWVRQEA-------QGARDCPECRRTFAEP-A----LAPSLKLANIVERYSSF
TRIM65       ------------------------------------------MAAQLLEEK---LTCAICLGLYQD----PVTLPCGHNFCGACIRDWWDRC-----------GKACPECREPFPDGAE----LRRNVALSGVLEVVRAG
TRIM68       --------------------------------------MDPTALVEAIVEE---VACPICMTFLRE----PMSIDCGHSFCHSCLSGLWEIPGESQ-----NWGYTCPLCRAPVQPR-N----LRPNWQLANVVEKVRLL
```

Supplementary Figure 3. Sequence alignment between PML- and TRIM- RINGs. The non-conserved F52/54 and L73 contact points are coloured in red. The conserved amino acids in the common to all TRIMs helix are coloured in purple. The invariant Zn binding residues are coloured in blue.

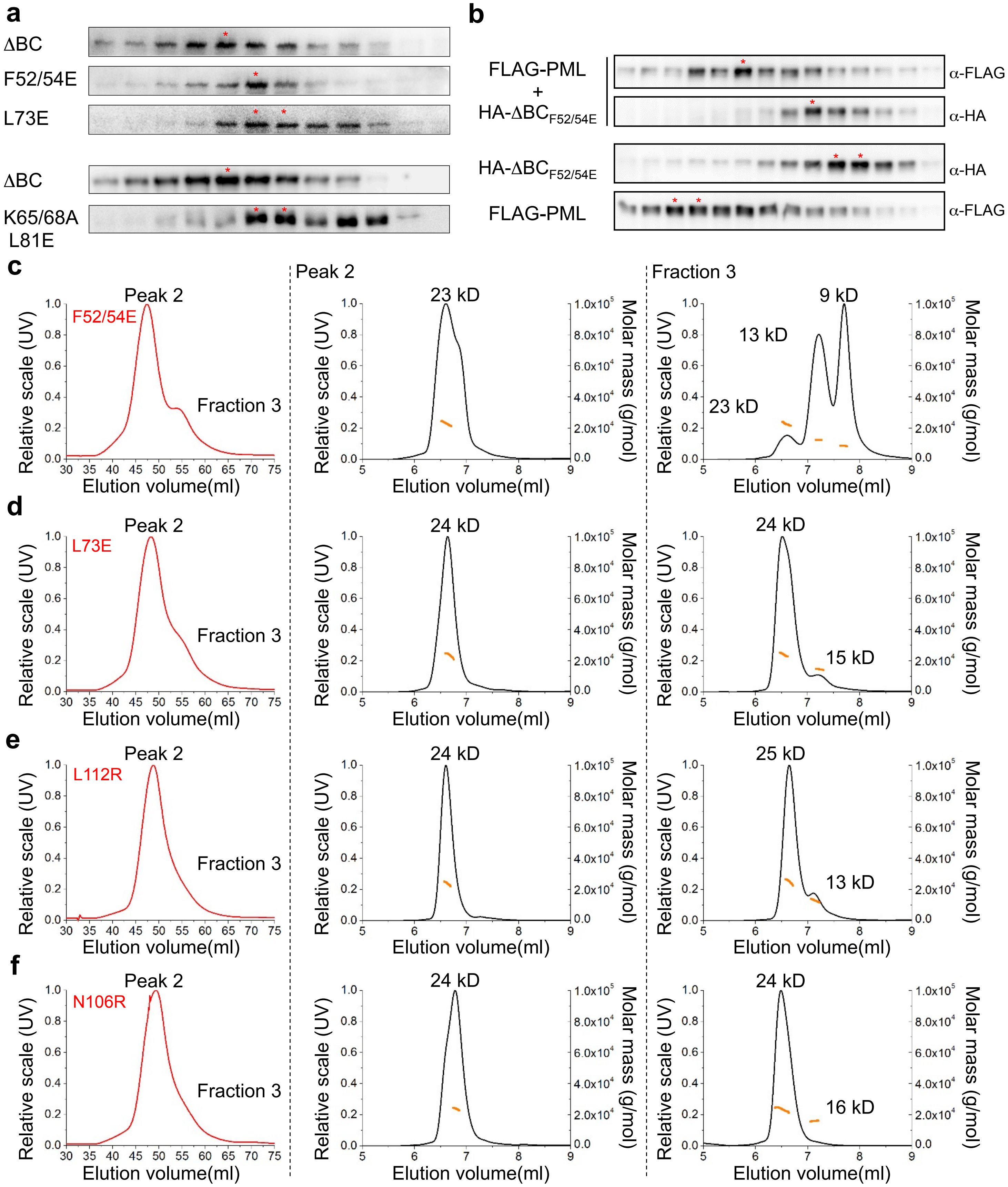

Supplementary Figure 4. Gel filtration and SEC-MALS analysis of PML RING$_{1\text{-}119}$ mutants. a) Gel filtration analysis of PMLΔBC (~42 kD), PMLΔBC $_{F52/54E}$, PMLΔBC $_{L73E}$ or PMLΔBC$_{K65/68A\ L81E}$. The peak positions are highlighted with "*". The PMLΔBC controls are paired with the respective mutants. b) Gel filtration analysis of Flag-PML together with HA-PMLΔBC$_{F52/54E}$. Gel filtration profiles of Flag-PML and HA-PMLΔBC$_{F52/54E}$ alone are also shown. c-f) Mutants F52/54E, L73E, L112R and N106R, respectively. Left panel, gel filtration analysis using S100 column. Middle and right panels, SEC-MALS characterization of peaks initially resolved in gel filtration. See also Figure 3.

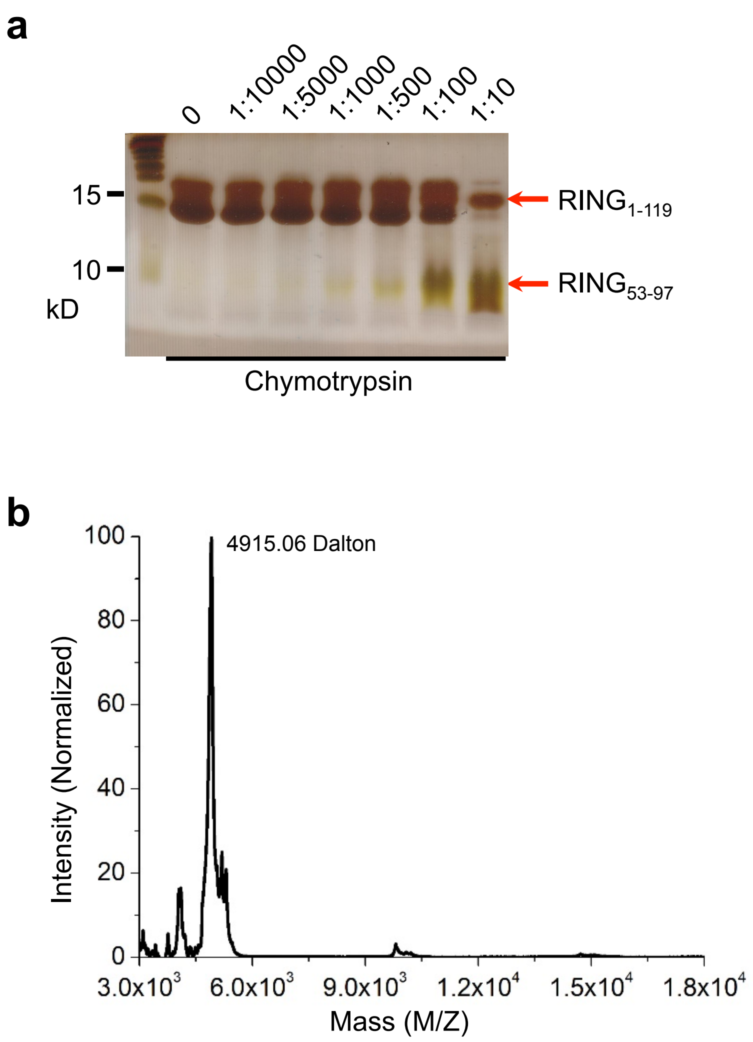

Supplementary Figure 5. Domain boundary of PML-RING. The stable fragment identified by the limited proteolysis treatment (a) was subjected to mass spectrometry analysis (b) and N-terminal sequencing analysis. See Fig. 3d for amino acid sequence of the fragment.

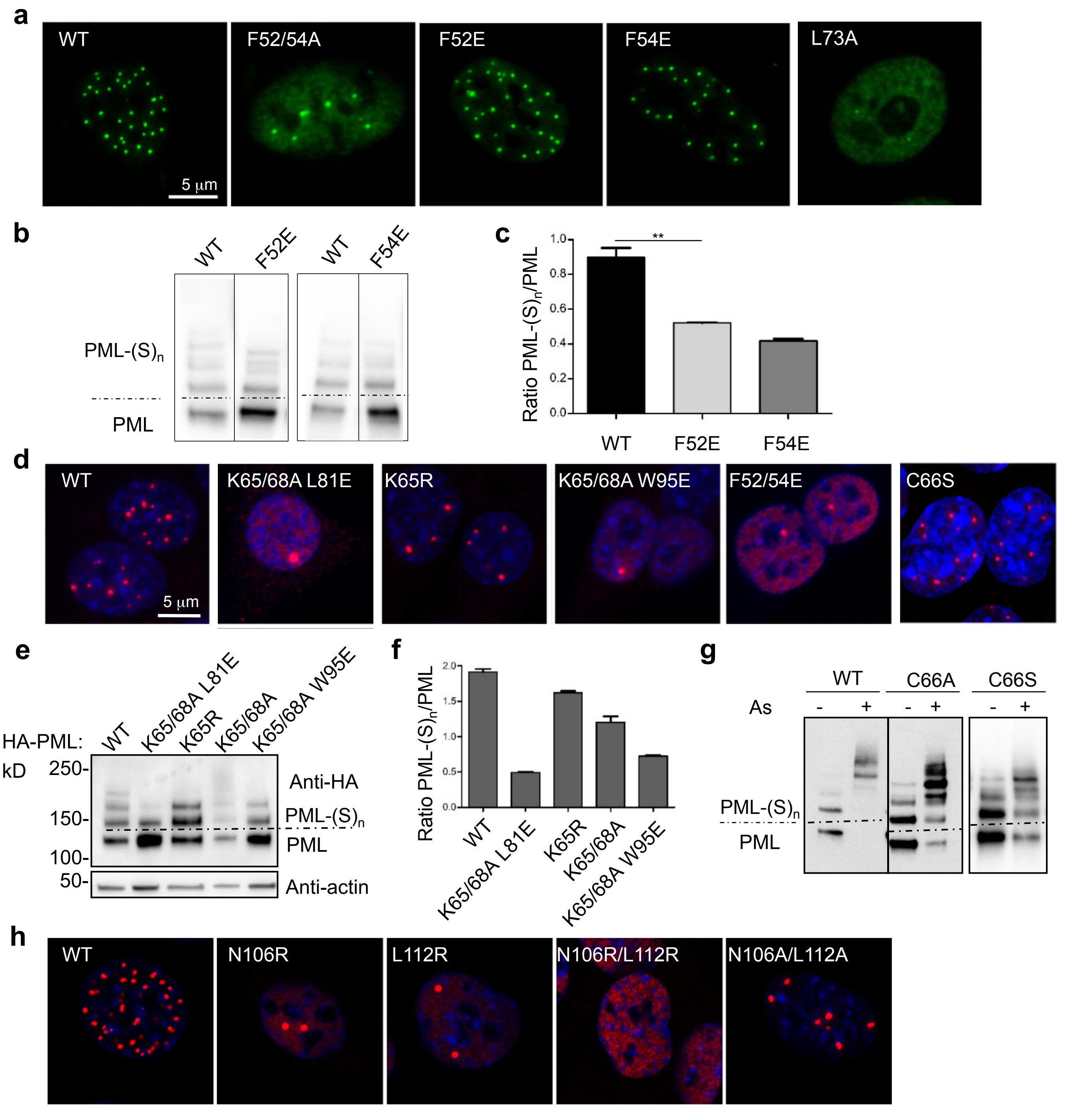

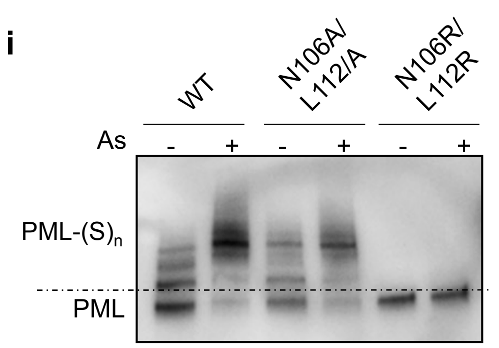

Supplementary Figure 6. Role of PML tetramerization in NB biogenesis and PML sumoylation. a) HA-$PML_{F52/54A}$, HA-PML_{F52E}, HA-PML_{F54E} and HA-PML_{L73A} were stably expressed in *pml*$^{-/-}$ MEFs and NBs formation were visualized using anti-HA antibody. b) Basal sumoylation was monitored by Western blot using HA-antibody. c) Statistical analysis of PML sumoylation. Values are means ± S.E. **, $p < 0.005$. d) NB formation of HA-PML_{WT}, HA-PML_{K65R}, HA-$PML_{K65/K68A,L81E}$, HA-$PML_{K65/K68A,W95E}$, HA-$PML_{F52/54E}$ and HA-PML_{C66S} was detected as above. e,f) The basal sumoylation of mutants detected using Western blot analysis (e) with quantification (f). Values are means ± S.E. g) Immortalized *pml*$^{-/-}$ MEFs stably expressing WT-PML, PML_{C66A} and PML_{C66S} were treated with/without As_2O_3 (10^{-6}M, 1 h) and extracts were analyzed by Western blot using anti-HA antibody. h) NB formation of HA-PML_{WT}, HA-PML_{N106R}, HA-PML_{L112R}, HA-$PML_{N106R/L112R,}$ and HA-$PML_{N106A/N112A}$. i) Immortalized *pml*$^{-/-}$ MEFs with WT-PML, $PML_{N106R/L112R}$ and $PML_{N106A/L112A}$ were treated with/without As_2O_3 (10^{-6}M, 1 h) and extracts were analyzed by Western blot.

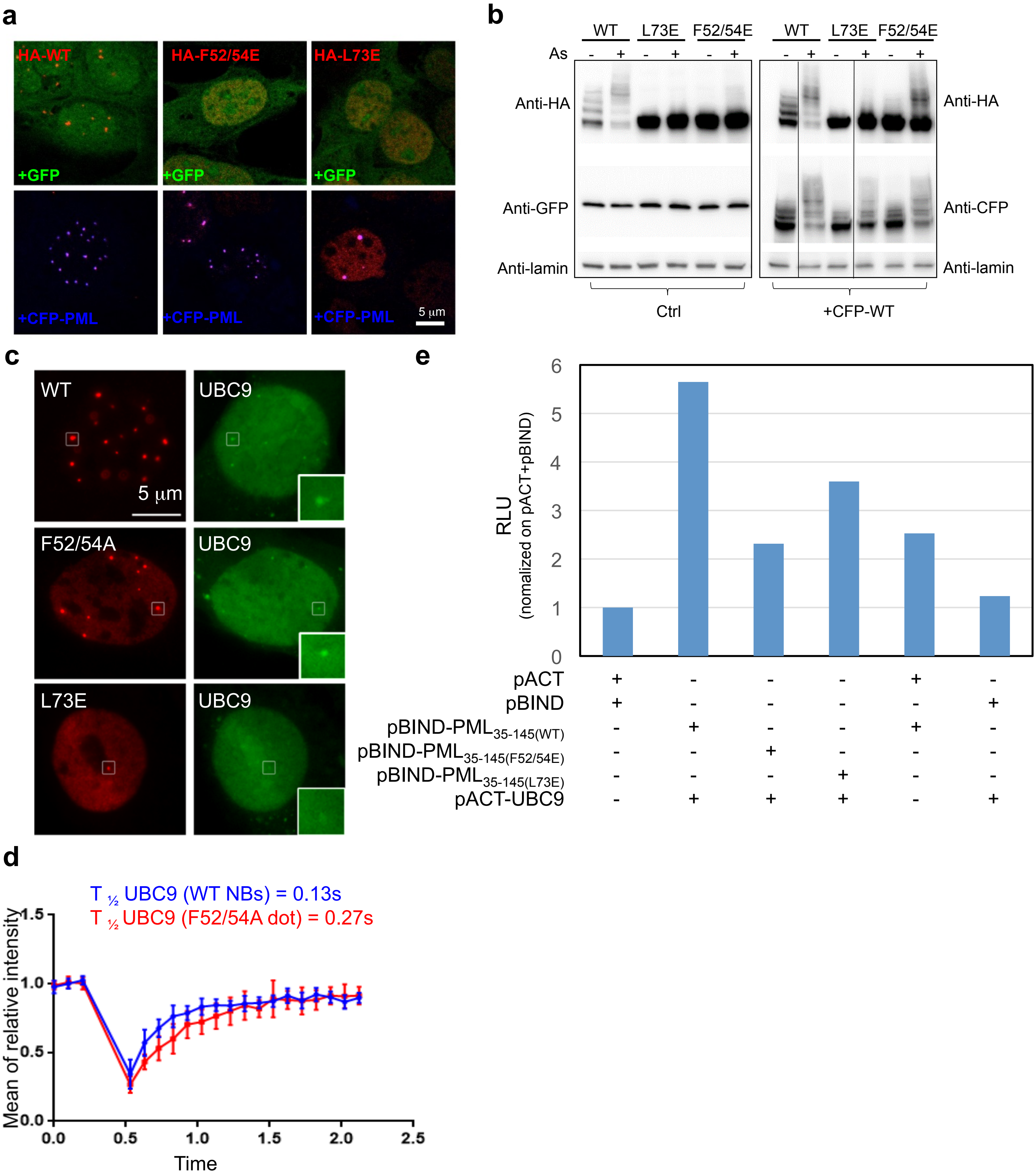

Supplementary Figure 7. PML RING tetramerization is important for UBC9 recruitment and sumoylation. a) Co-localization of HA-PML/$PML_{F52/54E}$/PML_{L73E} and CFP-PML. b) Complementation assays for $PML_{F52/54E}$ and PML_{L73E}. The *pml*$^{-/-}$ MEFs with stable expression of HA-wt PML, HA-$PML_{F52/54E}$ and HA-PML_{L73E} were stably transduced with CFP-wt PML. Sumoylation of the different PML constructs were probed using anti-HA or anti-GFP antibodies. Treatment with As_2O_3 (10^{-6}M, 1h) is indicated. Lamin control for loading. c) Co-localization of HA-wt PML (or mutant HA-$PML_{F52/54A}$, HA-PML_{L73E}) (red) and GFP-UBC9 (green) in *pml*$^{-/-}$ MEFs stably expressing HA-PML (or mutants) and GFP-UBC9, monitored by anti-HA antibodies and by GFP fluorescence. d) FRAP experiments for PML/UBC9 interaction. The boxed region containing one NB was selected in (c). GFP-UBC9 was bleached. Fluorescent recovery was quantified in three independent experiments. e) Mammalian two hybrid experiments revealing interactions between PML RING and UBC9.

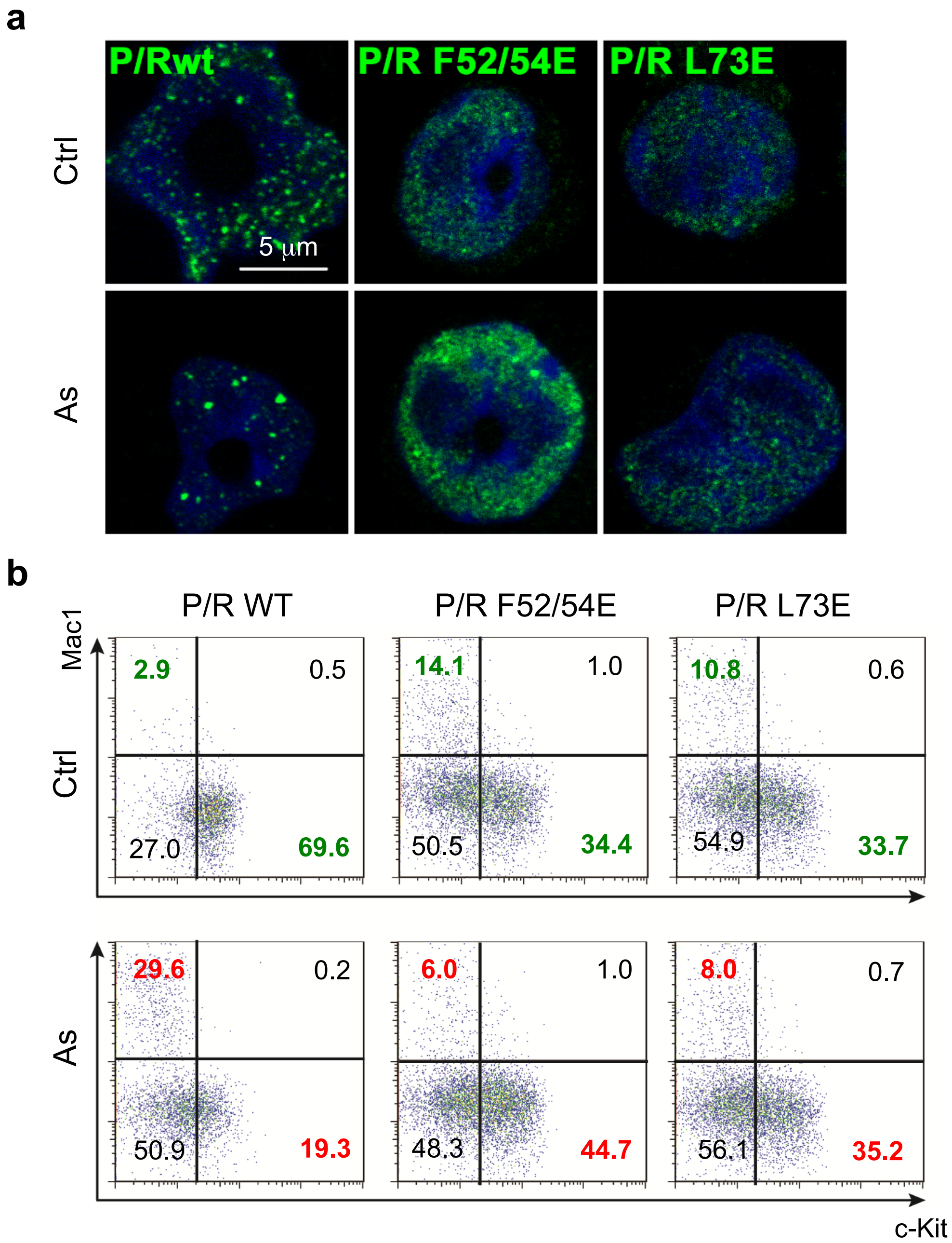

Supplementary Figure 8. RING tetramerization in arsenic response. a) Mouse hematopoietic progenitors containing PML/RARA and $PML/RARA_{F52/54E}$, $PML/RARA_{L73E}$ were treated with As_2O_3 (10^{-6}M, 1h). PML nuclear bodies were monitored by immuno-fluorescence using antibodies against human-PML. b) FACS profiles of PML/RARA- or mutant-transformed cells grown in methylcellulose in the presence or absence of 10^{-7}M As_2O_3 for 7 days.

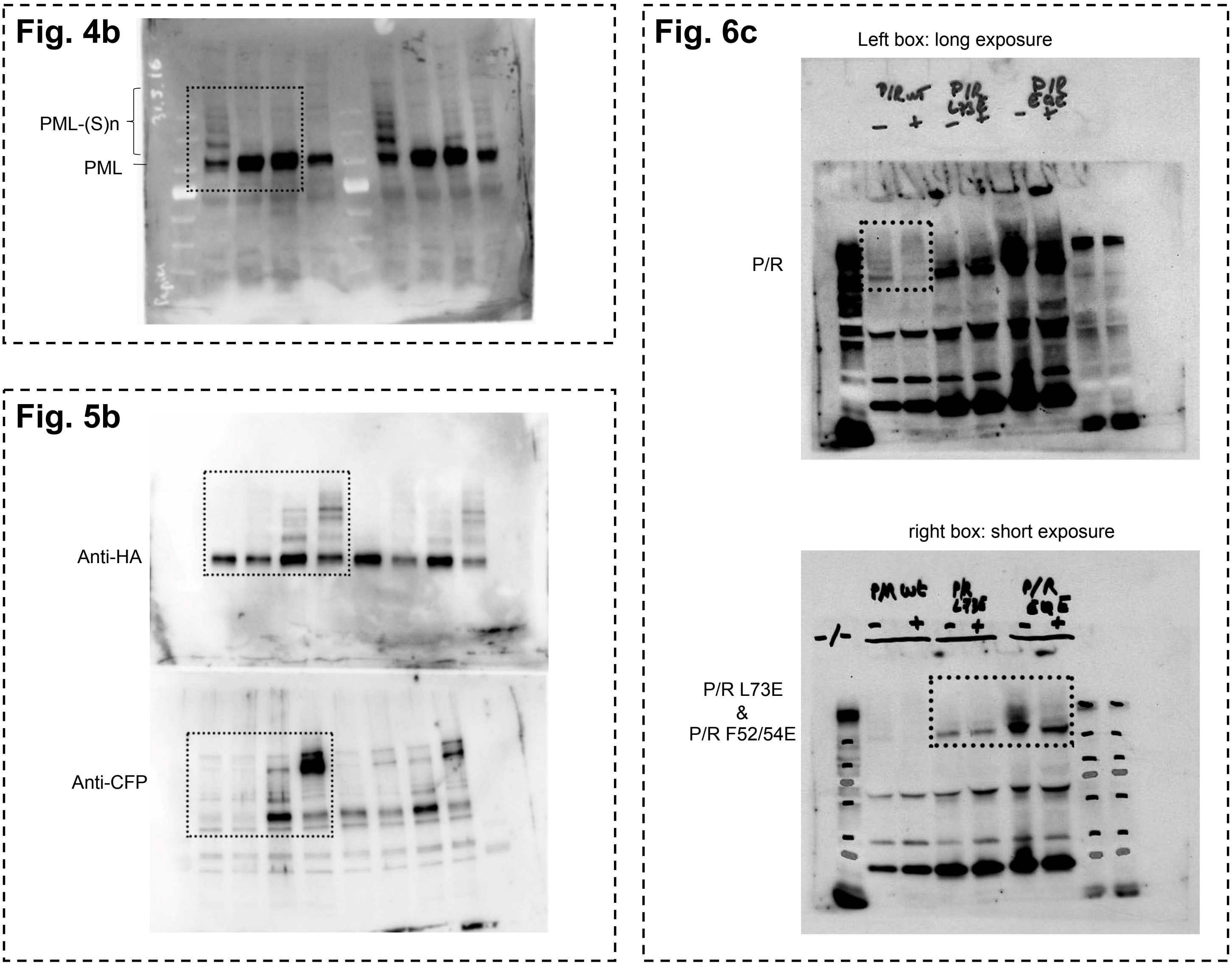

Supplementary Figure 9: Uncropped images of Western blot in Figure 4b, 5b and 6c.

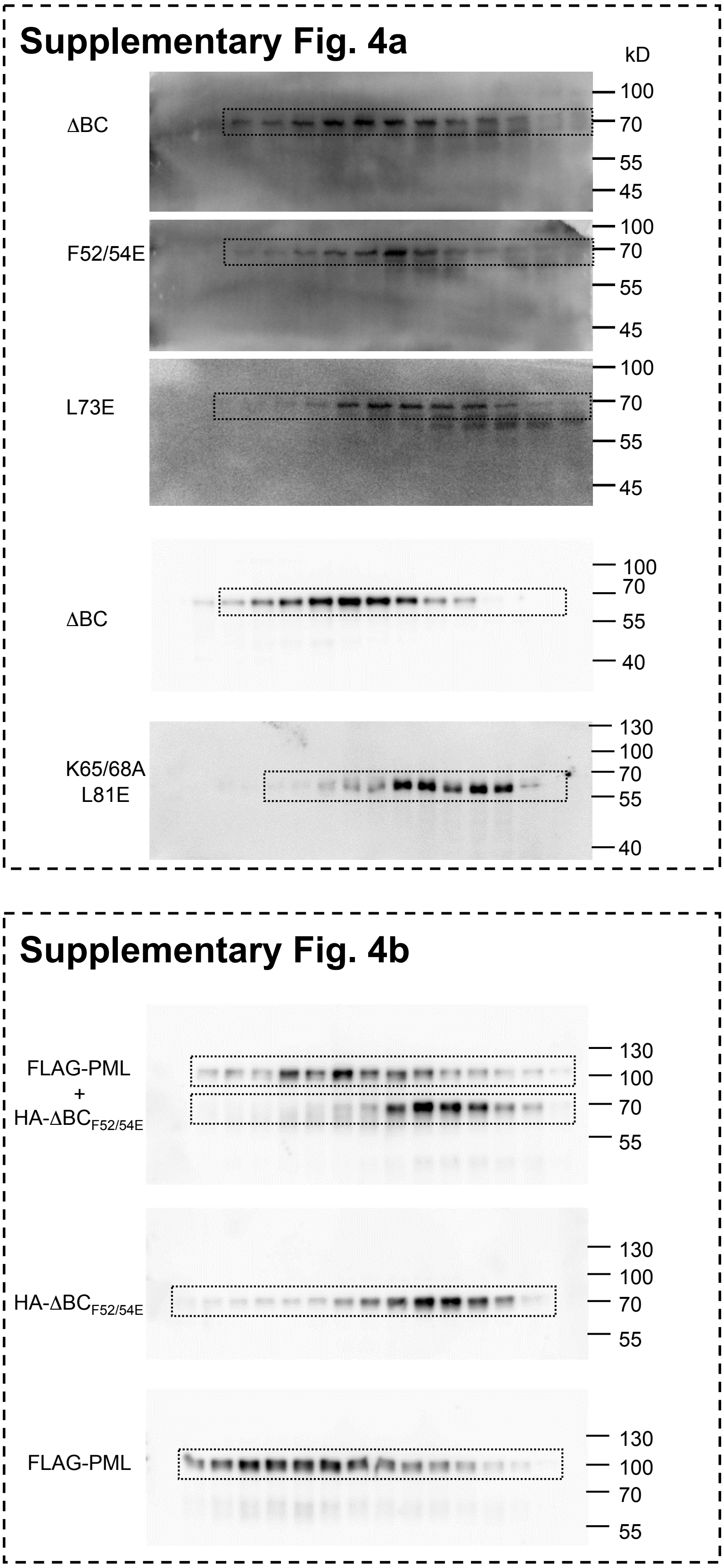

Supplementary Figure 10: Uncropped images of Western blot in Supplementary Figure 4a and 4b.

Supplementary Fig. 6b

WT
Long
exposure

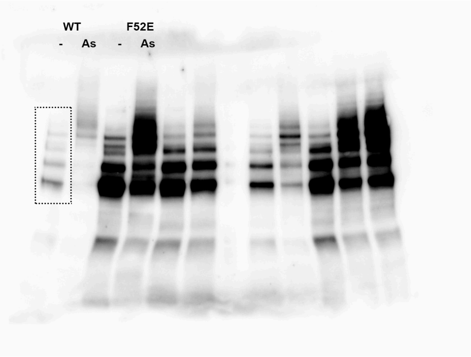

F52E
Short
exposure

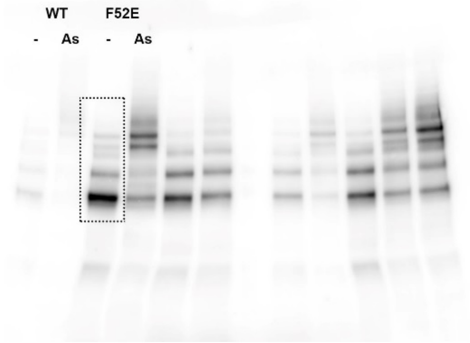

WT
&F54E

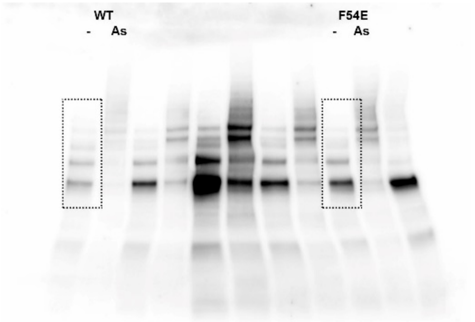

Supplementary Fig. 6i

Anti-HA

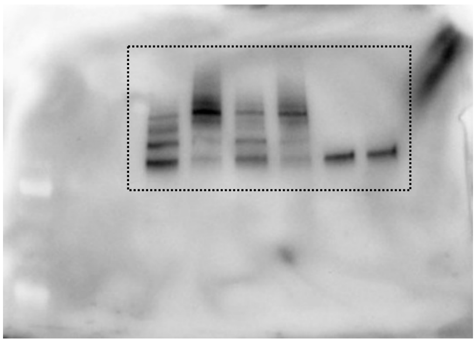

Supplementary Fig. 6e

Anti-HA

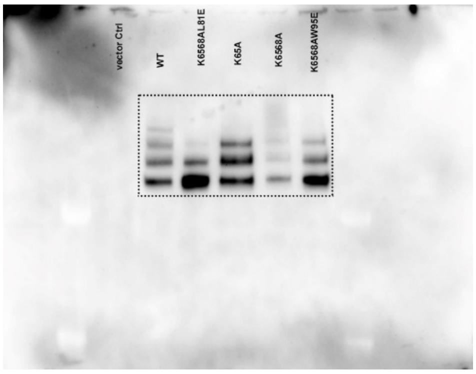

Anti-actin

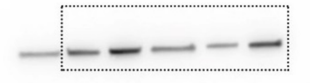

Supplementary Fig. 6g

Left box: long exposure

WT
&C66A

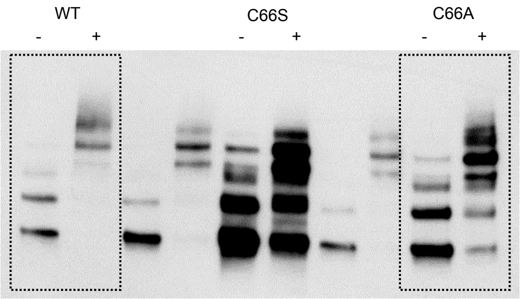

Rightbox: short exposure

C66S

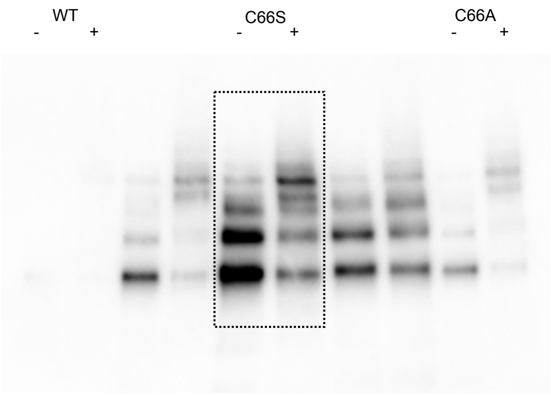

Supplementary Figure 11: Uncropped images of Western blot in Supplementary Figure 6b, 6e, 6g and 6i.

Supplementary Fig. 7b

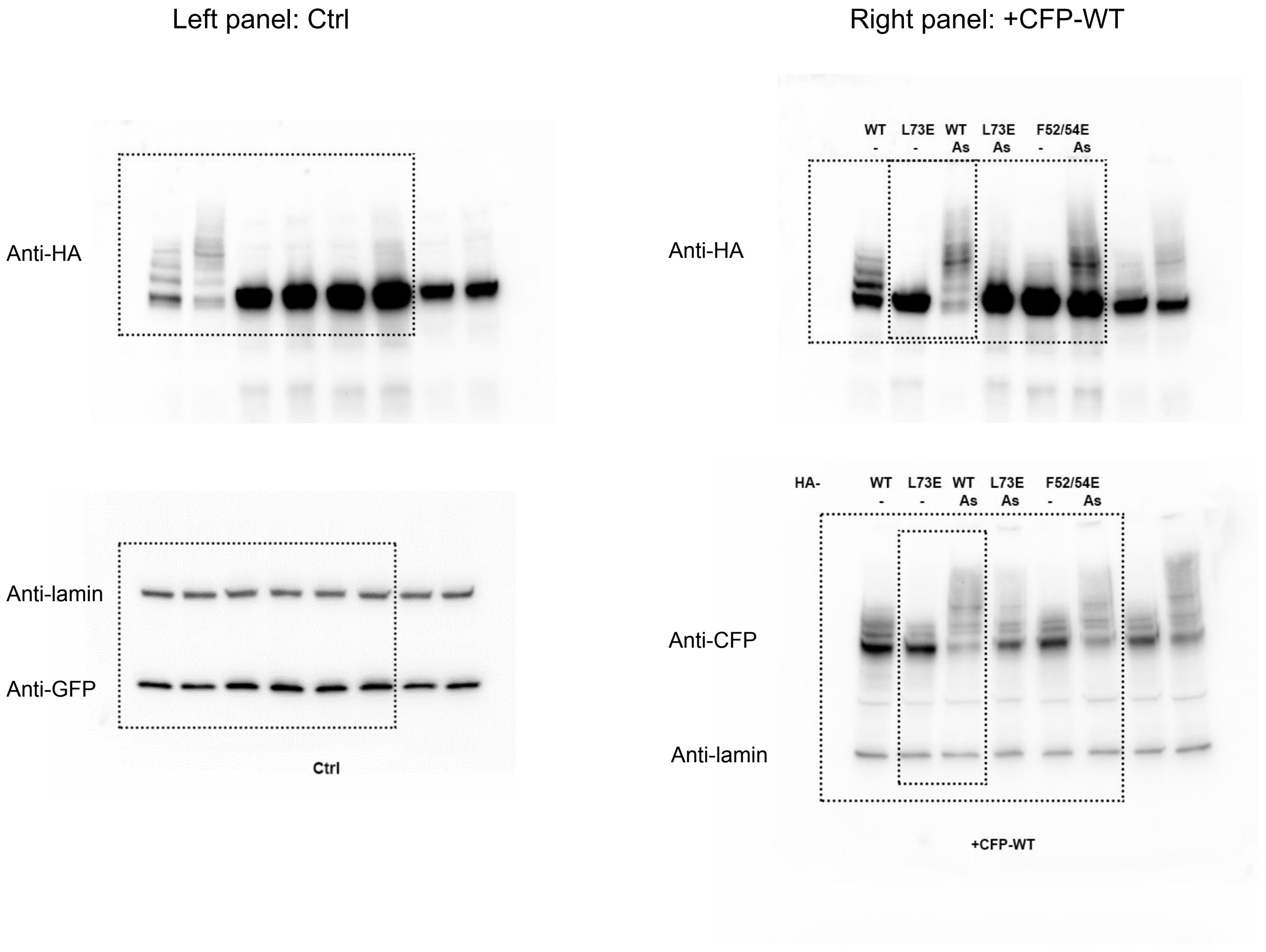

Supplementary Figure 12: Uncropped images of Western blot in Supplementary Figure 7b.

Supplementary Table 1 Primers used for sub-cloning

Plasmids	Primer Name	Primer Sequence (5' to 3')
pET32a-PML-RING$_{49-104}$	PML-49-BamHI-F	CGGGATCCgaggaggagttccagtttctgcgc
	PML-104-XhoI-R	CCGCTCGAGtcacagggcgggtgtgtctgc
pET32a-PML-RING$_{1-119}$	PML-1-BamHI- F	CGGGATCCatggagcctgcacccgc
	PML-119-XhoI-R	CCGCTCGAGtcagtacaccgacaggcgccg
pACT-PML	PML-BamHI-F	CGGGATCCGTatggagcctgcacccgc
	PML-XbaI-R	GCTCTAGActaaattagaaaggggtgggggta g
pBIND-UBC9	UBC9-BamHI-F	CGGGATCCGTatgtcggggatcgccctc
	UBC9-XbaI-R	GCTCTAGAttatgagggggcaaacttcttcg
pcDNA3.1(-)b-HA-PML	PML-EcoRI-kozak-HA-F	CGGAATTCGCCACCatgTACCCATACG ATGTTCCAGATTACGCTGAGCCTGCA CCCGCCC
	PML-BamHI-R	CCCGGATCCctaaattagaaaggggtgggggt ag
pBIND-PML-RING$_{35-145}$	PML-35-BamHI-F	CGGGATCCcccagccccagcccc
	PML-145-XbaI-R	GCTCTAGActgctcgcactcaaagcacca
pACT-UBC9	UBC9-BamHI-F	CGGGATCCGTatgtcggggatcgccctc
	UBC9-XbaI-R	GCTCTAGAttatgagggggcaaacttcttcg

Supplementary Table 2 Primers used for mutagenesis

Primer Name	Primer Sequence (5' to 3')
PML-L73E-F	cgaagctgctgccttgtgagcacacgctgtgc
PML-L73E-R	gcacagcgtgtgctcacaaggcagcagcttcg
PML-L73A-F	cgaagctgctgccttgtgcgcacacgctgtgc
PML-L73A-R	gcacagcgtgtgcgcacaaggcagcagcttcg
PML-F52/54E-F	ccccgcttcggaggaggaggagcaggagctgcgctgccagcaatgcc
PML-F52/54E-R	ggcattgctggcagcgcagctcctgctcctcctcctccgaagcgggg
PML-F52/54A-F	cgcttcggaggaggaggcccaggctctgcgctgccagcaa
PML-F52/54A- R	ttgctggcagcgcagagcctgggcctcctcctccgaagcg
PML-F52E-F	cgcttcggaggaggaggagcagtttctgcgctgcc
PML-F52E-R	ggcagcgcagaaactgctcctcctcctccgaagcg
PML-F54E-F	cggaggaggagttccaggagctgcgctgccagcaatg
PML-F54E-R	cattgctggcagcgcagctcctggaactcctcctccg
PML-K65/68A-F	ccaggcggaagccgcgtgcccggcgctgctgccttgt
PML-K65/68A-R	acaaggcagcagcgccgggcacgcggcttccgcctgg
PML-L81E-F	gtgctcaggatgcgaggaggcgtcgggc
PML-L81E-R	gcccgacgcctcctcgcatcctgagcac
PML-W95E-F	ctgccaggcgcccgagcccctaggtgca
PML-W95E-R	tgcacctaggggctcgggcgcctggcag
PML-N106R-F	cacacccgccctggatagggtctttttcgagagtctg
PML-N106R-R	cagactctcgaaaaagaccctatccagggcgggtgtg
PML-L112R-F	gataacgtctttttcgagagtcggcagcggcgcc
PML-L112R-R	ggcgccgctgccgactctcgaaaaagacgttatc
PML-N106R-L112R-F	ctttttcgagagtcggcagcggcgcctgt
PML-N106R-L112R-R	acaggcgccgctgccgactctcgaaaaag
PML-N106A-F	gacacacccgccctggatgccgtctttttcgagagt
PML-N106A-R	actctcgaaaaagacggcatccagggcgggtgtgtc
PML-L112A-F	gataacgtctttttcgagagtgcgcagcggcgcc
PML-L112A-R	ggcgccgctgcgcactctcgaaaaagacgttatc
PML-N106A-L112A-F	gatgccgtctttttcgagagtgcgcagcggcgcc
PML-N106A-L112A-R	ggcgccgctgcgcactctcgaaaaagacggcatc
PML-ΔBC(120-360)-F	cctgtcggtgtaccaggaggagcccc
PML-ΔBC(120-360)-R	ggggctcctcctggtacaccgacagg

Supplementary Table 3 SAXS data-collection parameters

**Data-collection parameters**	
Instrument	BL19U2 Beamline
Wavelength (Å)	1.03
q range (Å^-1)	0.01-0.25
Exposure time (sec)	1
Concentration range (mg ml^-1)	1-5
Temperature (K)	298
**Structural parameters**	
I (0) [from *P* (*r*)]	2.43*
R_g (Å) [from *P* (*r*)]	15.19 ± 0.21
I (0) [from Guinier]	2.43
R_g (Å) [from Guinier]	15.15 ± 0.21
D_{max} (Å)	49.87 ± 1.92
**Software employed**	
Primary data reduction	PRIMUS
Data processing	PRIMUS/GNOM
Computation of model intensities	CRYSOL
Estimation of oligomers in solution	OLIGOMER

R_g: Radius of Gyration, D_{max}: maximum particle size
**I* (0) [sample concentration (mg ml^-1)] ^-1
